# Immediate and Sustained Effects of Neurofeedback and Working Memory Training on Cognitive Functions in Children and Adolescents with ADHD: A Multi-Arm Pragmatic Randomized Controlled Trial

**DOI:** 10.1177/10870547211063645

**Published:** 2022-01-16

**Authors:** John Hasslinger, Ulf Jonsson, Sven Bölte

**Affiliations:** 1 Center of Neurodevelopmental Disorders (KIND), Centre for Psychiatry Research, Department of Women’s and Children’s Health, Karolinska Institutet, Sweden; 2 Child and Adolescent Psychiatry, Stockholm Health Services, Region Stockholm, Stockholm, Sweden; 3 Department of Neuroscience, Child and Adolescent Psychiatry, Uppsala University, Uppsala, Sweden; 4 Curtin Autism Research Group, Curtin School of Allied Health, Curtin University, Perth, Australia

**Keywords:** neurofeedback, biofeedback, ADHD, slow cortical potentials, live *Z*-score, working memory training, neuropsychology, cognition, intervention, training

## Abstract

**Objective:**

To evaluate the effects of neurocognitive training methods on targeted cognitive functions in children and adolescent with ADHD.

**Method:**

A pragmatic four-arm randomized controlled trial compared two types of neurofeedback (Slow Cortical Potential and Live *Z*-score) and Working-memory training (WMT) with treatment as usual. *N* = 202 participants with ADHD aged 9 to 17 years were included. A battery of cognitive function tests was completed pretreatment, posttreatment, and after 6-months.

**Results:**

The effects of WMT on spatial and verbal working-memory were superior to neurofeedback and treatment as usual at posttreatment, but only partially sustained at follow-up. No other consistent effects were observed. We found no clear indications that effects were moderated by ADHD presentation, ongoing medication, age, or sex.

**Conclusion:**

The sustained effects of neurocognitive training on cognitive functioning in children and adolescents with ADHD may be limited. Future research should focus on more personalized forms of neurocognitive training.

## Introduction

With a prevalence of 5% to 7% (Polanczyk et al., 2014; Thomas et al., 2015), ADHD is one of the most common mental health conditions of childhood (Thapar, 2018). While age inappropriate behavioral patterns of inattention, hyperactivity and impulsivity constitute the defining features of ADHD (American Psychiatric Association, 2013; World Health Organization, 2004), various cognitive alterations are also characteristic of the condition (Bolte et al., 2018; Rubia, 2018). Deficits in executive functions are central in ADHD, affecting verbal and spatial working memory, planning, attention, and vigilance (Sergeant, 2005; Willcutt et al., 2005). Other prominent cognitive impairments include temporal processing, inhibition (Sonuga-Barke et al., 2010), emotional dysregulation (Shaw et al., 2014), the preference of small immediate rewards (Marx et al., 2021), and impaired overall decision making (Sonuga-Barke et al., 2016). Recent meta-analytic work, mapping and evaluating a broad array of cognitive challenges in ADHD, suggest moderate functional alterations in domains such as working memory, inhibition, cognitive flexibility, vigilance, and reaction time variability (Campez et al., 2020; East-Richard et al., 2020; Pievsky & McGrath, 2018). In addition, a recent meta-analysis suggest pronounced time perception deficits in children and adolescents with ADHD (Zheng et al., 2020).

Importantly, ADHD is a heterogeneous condition and patterns of cognitive functioning may vary substantially between individuals. Performance-based cognitive tests tend to show inconsistent results, with only subsamples underperforming across a multitude of tasks (Mahone & Denckla, 2017). While it has been suggested that ADHD is more precisely subtyped based on executive function profiles rather than by the diagnostic division (Roberts et al., 2017), the diagnostic ADHD presentations may also differ in their cognitive profiles. Inhibitory difficulties have been reported to be more prevalent in the combined presentation, while the predominantly inattentive presentation has been linked to motivational problems and under-arousal (Diamond, 2005; Pachalska et al., 2014).

Cognitive alterations have been associated with multiple real-life adversities and mental health issues, including academic underachievement (Fried et al., 2016), anxiety and depression (Hatch et al., 2007), violent offenses (Zou et al., 2013), binge-drinking (López-Caneda et al., 2017), and social functioning (Dawson et al., 2012). Therefore, interventions addressing cognitive functions are of clinical relevance. In addition to effects on the defining ADHD core behavioral symptoms (Cortese, 2020), stimulants have also been reported to enhance cognitive functions in both children and adolescents, including improved response inhibition (Standardized Mean Difference, SMD: 0.41), executive and non-executive memory (SMD: 0.26 resp 0.60), reaction time (SMD: 0.24), and reduced reaction time variability (SMD: 0.62) (Coghill et al., 2014). Nonetheless, side effects such as appetite suppression, insomnia, nausea, abdominal pain, and headaches are relatively common and may lead to inconsistent treatment adherence and discontinuation (Frank et al., 2015; Sharma & Couture, 2014). Long-term side effects, in particular height suppression (Swanson et al., 2017) and cardiovascular functioning (Smith et al., 2010) have also been reported, emphasizing the importance of non-pharmacological treatment alternatives.

Neurocognitive training methods like neurofeedback (NF) and working memory training (WMT) are non-invasive treatment options, which in recent decades have received increased research attention (Goode et al., 2018; Hodgson et al., 2014). NF aims to enhance cortical functioning by training the brain’s electrical activity through operant learning and thereby affect the brains ability for self-regulation, that is the flexibly to adapt brain activity to more effectively meet the changing demands of the environment (Arns et al., 2014). Over time, the training can lead to neurophysiological changes in the brain (Lévesque et al., 2006), which in its turn might lead to improvement in ADHD symptoms. WMT is a computerized intervention that targets different working memory functions. The gamified training utilizes adaptive difficulty levels and is performed on a daily basis to enhance working memory capacities (Klingberg et al., 2002, 2005). Most NF studies have focused on the effects on behavioral core symptoms, indicating improvements when rated by parents but not for teacher-ratings (Cortese et al., 2016). Effects seem to be sustained and possibly grow over time, when compared to non-active controls (Van Doren et al., 2019). The impact of NF and WMT on cognitive functions has been examined to a lesser extent, and the available research has yielded mixed results. While some studies found NF to improve executive functions (Minder et al., 2018; Steiner et al., 2014) and working memory (Dobrakowski & Łebecka, 2020), others failed to show such effects (Bink et al., 2014; Vollebregt et al., 2014). A recent meta-analysis of non-pharmacological interventions’ impact on cognitive functions found medium sized effects of *d*′ = 0.61 for NF, and *d*′ = 0.45 for other forms of neurocognitive training (Lambez et al., 2020). Pooled results for all non-pharmacological interventions showed effect sizes ranging from *d*′ = 0.40 for working memory to *d*′ = 0.69 for inhibition. As for WMT, meta-analyses mainly indicate short-term (Melby-Lervåg & Hulme, 2013; Shipstead et al., 2012) and near-transfer effects (i.e., improved working memory but no effects on untrained components) (Kassai et al., 2019).

Given these overall inconclusive findings regarding the effects of NF and WMT on cognitive functioning in ADHD, more comparative and ecologically valid research is needed to evaluate the clinical usefulness of neurocognitive training for children and adolescents with ADHD. Herein, we present secondary outcomes from a pragmatic, open-label trial, examining the effects of WMT and two different forms of NF (a well-researched and a newer, less researched protocol) on measures of working memory, time perception, inhibition, and inattention. To further increase the clinical usefulness of the results, we investigated to what extent results were sustained over time and whether the outcomes were moderated by ADHD presentation, medication status, age, and sex.

## Method

### Trial Design

This work is part of the KITE study (NCT01841151) (Hasslinger et al., 2016), a pragmatic single site four-arm randomized controlled open-label trial of neurocognitive training interventions in children and adolescent with ADHD, conducted at an outpatient clinical research unit in Stockholm, Sweden. Participants were recruited either via self-referral or by clinical referral by child and adolescent psychiatry and pediatrics, and enrolled continuously between 2013 and 2019. The results presented here were based on secondary outcome measures, for which no explicit hypotheses were specified in advance. Primary outcomes (i.e., ADHD core symptoms) are reported elsewhere (Hasslinger et al., 2021). The study was approved by the Ethical Review Board in Stockholm. Written informed consent was obtained from all participants’ legal caregivers, and assent from the participants.

### Participants

The sample consisted of *N* = 202 (49 girls, 153 boys) children and adolescents aged 9 to 17 years, with a previous primary community diagnosis of ADHD, combined type or inattentive type (American Psychiatric Association, 2013; World Health Organization, 2004) form the Swedish public healthcare system according to regional assessment guidelines (Axén et al., 2010). Comorbidity with other common diagnoses (e.g., autism spectrum disorder) was not an exclusion criterion, with the exception of acute conditions that required prioritized clinical attention (e.g., depression with suicidal thoughts, severe eating disorders). Insufficient Swedish language proficiency and IQ < 80 were also exclusion criteria. Ongoing pharmacological treatments were allowed but had to remain unchanged in type and dosage throughout study participation.

### Procedure

Following informed consent, potential participants were evaluated for inclusion- and exclusion criteria. If additional information was needed in order to rule out intellectual disability, a complementary assessment was conducted using the Wechsler Intelligence Scale for Children or Adults fourth editions (Wechsler, 2009, 2011). Each assessment point consisted of a full day of testing, including EEG-assessments and cognitive tests. At the baseline assessment, the Kiddie Schedule for Affective Disorders and Schizophrenia interview (Kaufman et al., 1996) was conducted with a parent or other caregiver, in order to confirm the ADHD diagnosis and evaluate excluding psychiatric conditions. Both the cognitive testing and the interview were conducted by a clinical psychologist or a supervised student in clinical psychology. A 48-hour wash-out period prior to each assessment point was implemented for stimulant medicated participants. Following the baseline assessment, the active conditions underwent daily sessions (5 sessions/week) during five subsequent weeks (25 sessions in total). Missed sessions, due to illness or schedule conflicts, were replaced, postponing the post-assessment. However, the maximum training period length was 7 weeks in order to maintain the high session intensity, and for scheduling purposes. All subjects, participating in the post-assessment, completed at least 23 sessions. NF sessions lasted for around 60 minutes, while WMT sessions usually lasted around 40 minutes. However, WMT did not require any preparation (i.e., electrode placement) leaving the length of the active training component similar for the training methods. The training period was followed by the post assessment (T2) within a week after completing the training sessions. Two additional booster sessions were conducted shortly before the 6-month follow-up assessment (T3). Participants earned points each session (not performance based, see Hasslinger et al., 2021), toward a reward gift certificate SEK 200 (USD ~22) that was paid at post assessment. An additional certificate worth SEK 500 (USD ~55) was rewarded after completing follow-up assessments.

### Randomization

The first 100 participants were randomized to one of the four conditions (one of two types of NF, WMT, or TAU) via a prepared dual-lane number sequence. One lane did not include WMT, and was utilized for participants who had partaken in WMT in school or at home before entering the study. Allocation was determined by the date of the finalized eligibility assessment. The subsequent participants were allocated via a list generator at random.org, that included the remaining empty spots (based on 50 per intervention minus already allocated). The final five participants were randomized simultaneously in order to avoid predictability.

### Staff

The interventions (NF and WMT) were conducted by 19 trainers (3 clinical psychologists, 4 research nurses, and 12 supervised students in clinical psychology). Trainers had been provided with inhouse training by experienced trainers, including practice in all training methods. Trainers were then supervised by experienced trainers during their first sessions, before being permitted to conduct sessions independently. Furthermore, a step-by-step guide for each intervention was developed, and all trainers communicated frequently with each other, for further stringency. Most trainers conducted both NF and WMT training. The standardized cognitive tests and psychiatric scales were administered by the psychologists or psychology students trained in psychological assessment.

### Interventions

#### Slow cortical potential training (SCP)

Slow cortical potentials are event-related potentials, measured as slow shifts in the bioelectrical activity in the brain. They are characterized by negative or positive shifts lasting from 300 msec. to several seconds (Birbaumer et al., 1990). These shifts are assumed to reflect states of either increased cortical excitability (negative shifts) or reduced excitability/inhibition (positive shifts) and there are indications that the regulation of slow cortical potentials is altered in children with ADHD (Gevensleben et al., 2014). SCP is intended to increase control over these shifts, ostensibly improving self-regulation and reducing behavioral symptoms of ADHD.

SCP was conducted using a TheraPrax™ (NeuroConn, Germany). Ag/AgCl electrodes were used, with four electrodes placed around the eyes, measuring the electrooculogram. The active electrode was placed at Cz, while the reference and ground electrodes were placed on the mastoids. Impedance was kept under 5 kΩ. Once the electrodes were placed, a calibration for the online eye movement correction was conducted. Each SCP session consisted of 144 trials lasting 10 seconds (2 seconds baseline, 8 seconds feedback) split into 4 blocks of 36 trials. During each trial, the participant was presented with a triangle on a computer screen, that pointed upwards or downwards. Another object on the screen moved across the screen, from left to right. The task was to move this object in the same direction as the triangle, by regulating the slow cortical potentials. When regulating successfully, a star was shown onscreen. So-called transfer trials did only show the triangle, and if successful, the star. Their purpose was to facilitate self-regulation without the need of real-time feedback. Transfer trials constituted 20% of all trials during week 1, 40% during week 2, and 50% for the remaining training period. Participants who successfully self-regulated during transfer trials of the last three sessions, based on the average µV value of the last 3 seconds per trial, were categorized as learners. This meant generating a negative µV value on average for the activation trials, and a positive µV value for the deactivation trials. It shall be noted that no additional manual artifact corrections were conducted.

#### Live Z-score training (LZT)

Quantitative electroencephalographic (QEEG) transforms the EEG measures to *z*-scores (Wigton & Krigbaum, 2015), and allows to compare the individuals EEG activity to a norm-referenced population (database). In LZT, real-time estimates of these measures are used to provide feedback to the participant during training in an attempt to normalize EEG activity (Collura, 2016). As there is considerable variation in LZT regarding which parameters are used (e.g., amplitude, power or coherence), how ranges are defined, and how conversion of *z*-scores into feedback signals is done (Collura, 2016), it is considered a non-standard protocol (Thibault & Raz, 2017). While LZT is popular, and applied by many private treatment providers due to its easy implementation, it is lacking support from peer-reviewed research (Coben et al., 2019).

LZT was conducted using an AtlantisII™ (Brainmaster Inc., Bedford, OH, USA), with AgCl snap connectors, and utilization of the ANI database (Applied Neuroscience Ltd, Florida, USA). Each session consisted of two blocks with 20 minutes continuous feedback. Electrode placement were at C3 and C4 for the first block, and Fz and Cz during the second block. The reference was set at the left earlobe, and ground was first at Cz and at C3 for block 2. Impedance was kept under 5 kΩ. A so-called PZOK protocol was utilized, that measured all available parameters (e.g., absolute/relative power, asymmetry, coherence, etc.) and calculated an overall percentage of the parameters *Z*-values that were within the ±1.5 *SD Z*-score range. The percentage-threshold was adjusted manually, targeting a reward rate of around 60% to 70%, thereby keeping the difficulty at a reasonable level for each participant.

During the first 5 to 10 minutes of each session, feedback was given using BrainCells™ (BrainMaster Ltd.), a game where “brain cells” appear faster and smoother on the screen, depending on the participants performance (matching the set *Z*-score percentage). Success was further reinforced via auditory effects. Thereafter, participants could choose visual stimuli from Netflix™ or Youtube™ and a transparent dimmer window (Tor Ghai, Stockholm, Sweden) was placed on top of the stimuli, which turned opaque when the participant deviated from the targeted *z*-score percentage. Participants were instructed to sit still during training, but no other specific instructions were provided. Overall, sessions lasted around 60 minutes.

#### Working memory training (WMT)

For WMT we used a computerized software program with visuospatial and auditory tasks called MinneslekFlex™ (www.flexprogram.org), a training tool commonly used in school settings across Sweden (Von Greiff et al., 2012). The participants could choose between a Junior and a Senior version that differed on the thematic content while sharing the same structure. In both versions, every session consisted of 6 different exercises with 12 trials each. In two exercises had auditory-stimuli, and two of the four visual-stimuli exercises included distracting elements such as movement of the stimuli. The level of difficulty was automatically adjusted once enough consecutive responses were correct (increase) or wrong (decrease). The program is comparable with CogMed (Roche & Johnson, 2014).

#### Treatment as usual (TAU)

All participants, including the participants randomized to TAU, were instructed to not change ongoing treatments for ADHD, nor start new treatments, until follow-up. No additional restrictions were imposed. Data about ongoing pharmacological treatment were collected, but not for other interventions including dietary supplements. In accordance with regional guidelines for treatment of ADHD, many of the children’s parents underwent psychoeducational parent group-training prior to study inclusion (Axén et al., 2010). No psychological treatments for ADHD were reported.

### Outcomes

#### Working memory

Verbal working memory, was measured via face-to-face administered forward and backward versions of Digit Span and Letter-Number Sequencing, from the WISC-IV/ WAIS-IV (Wechsler, 2009, 2011). Digit span constitutes of sequences of verbally presented numbers which the participant is required to repeat. Only the forward and backward portions of the test were included in the analysis, as sequencing is only available in the WAIS-IV version. Letter-Number Sequencing, entails both numbers and letters presented verbally. The task is to repeat the numbers and letters separately, sorted in numeric or alphanumeric order. The scaled scores (10 ± 3) of the subtests were used in the analyses.

Spatial working memory was measured via the forward and backward versions of block-tapping task from WISC-IV-integrated/ WAIS-III NI (Wechsler, 2004; Wechsler et al., 2004), and the “Find the phone” task. During block-tapping, the test administrator points at cubes on a board in a specific sequence. The participant is instructed to subsequently point at the cubes in the same order. Although the test administration is identical when using the WISC-integrated version (for subjects <16 years) or the WAIS-III-NI version (for subjects ≥16 years), the available scaled scores are not comparable as scaled scores are either separated by direction (WISC-IV-integrated) or only available as a joint score (WAIS-III-NI). Raw scores (maximum 19) were therefore used. The “Find the phone” task is a generic version from the spatial working memory task included in the Cambridge Neuropsychological Test Automated Battery (CANTAB), and has been used in previous studies (Owen et al., 1990; Sjöwall et al., 2013). The test is computerized and measures the participant’s ability to retain visual-spatial memory of a number of phones displayed on a computer screen. Two test performance outcomes were collected: between-search errors (BSE) and within-search errors (WSE). BSE occur when clicking on a phone that has already been answered in a previous trial in the same level, while WSE occur when clicking on a phone multiple times in the same trial. Two levels with 4, 6, and 8 appearing phones each were administered. The total number of WSE and BSE were defined as outcome measures. With the exception of the forward modalities of digit span and block-tapping, we regarded all other task as measures of aspects of executive memory (i.e., requiring some sort of cognitive control, as compared to simple recall).

#### Time perception

The Time Anticipation (visual cueing) and Tapping tasks (auditory cueing), previously used by Toplak and Tannock (2005), were applied to assess time perception. Time Anticipation is a computerized time perception and impulsivity task. It is framed by a short story, where participants have to beam oxygen over to a spaceship, in order to save the crew. As soon as the ship becomes visible on the screen, the participants have to press the left mouse button. The spaceship appears with the same time interval during each trial. However, after 10 trials the spaceship becomes invisible, and the participant has to anticipate when the invisible ship is appearing, and click within a 750 ms window. Feedback is given in both visible (cued) and invisible (uncued) trials, notifying the participants when they are on time, too early, or too late. Two versions were conducted, one with a response rate of 400 m and a second for 2,000 ms. The hit rate for correct and too early responses during uncued trials was defined as the outcome measure of interest. Tapping is a computerized time and frequency critical motor control task, where a tone is presented every 1,200 ms for 15 trials. The participant is asked to tap the left mouse button at the same pace. After initial cued trials, the participant is asked to continue tapping at the same pace for 41 uncued trials. This is followed by a second run, consisting of 15 cued and another 41 uncued trials. Mean tapping rate and standard deviation (*SD*) were calculated for the last 40 uncued trials per run. The coefficient of variability was calculated via the subjects’ *SD*/mean tapping rate × 100 and served as outcome measure. Values that deviated more than three *SD*s from a run’s mean were deemed to be caused by interference (e.g., due to sneezing, or other interruptions) and consequently deducted.

#### Inhibition and attention

The Conner’s Continuous Performance Test-II (CPT-II) was used to measure inhibition and attention functions. The CPT-II is a widely applied computerized task with incremental clinical utility (Tallberg et al., 2019). It generates multiple outcomes including inattentiveness, impulsivity, sustained attention, and vigilance. The participant is instructed to press the left mouse button as soon as a letter appears on the screen, but needs to abstain from clicking when the letter is an “x.” Inhibition is mainly measured via commission errors, which occur upon false responses to the non-target “x.” Missing to correctly respond to targets, omission errors, reflect sluggish attention. The reaction time and standard error (SE) also indicate inattentiveness. The normative *t*-values (50 ± 10) for the above variables were used as the outcome measure, along with the CPT-II ADHD-index which provides an overall ADHD response pattern in form of a likelihood percentage. To facilitate comparability with other studies, raw scores, including reaction times separated for Hits and Commissions, are provided in the supplement (Supplemental Table S1a).

### Sample Size and Statistical Methods

In accordance with the intention-to-treat principles, primary and secondary analyses included all randomized participants for whom data were available at baseline. The number of participants per arm was set in advance to 50, providing a power (1-beta) of >0.99 for a large effect and 0.80 for a medium effect at alpha = 5% and an expected attrition rate of 10% (G*Power 3.1.7). The originally planned MANOVA (Hasslinger et al., 2016) was replaced by mixed-effect linear modeling (random regression), which currently is the preferred choice for analysis of repeated-measures data (Gueorguieva & Krystal, 2004). The model was specified by using time (baseline, posttreatment, follow-up), treatment group, and the time by group interaction as fixed effects, as well as a random intercept for each participant. A separate model was run for each comparison. The treatment effect (time by group interaction) was expressed as the group difference in the change of least-squares mean scores from baseline to posttreatment/follow-up. No adjustments for multiplicity were applied. For significant effects, potentially moderating effect of age (<13 vs. ≥13 year), sex, ADHD presentations (combined vs. predominantly inattentive subtype), and ADHD-medication status (medicated or non-medicated), were explored by including the three-way interaction of time by group by moderator, all main effects, and all lower-order interactions terms in the model. Whenever a significant three-way interaction was found, stratified analyses were conducted. In case a significant difference between any of the groups was present at baseline, potentially moderating effects of the variable in question were explored for all outcomes. For SCP we also explored differences between learners/non-learners, by comparing the outcome of those who were classified as learning to those who were not. Between-groups effect sizes were estimated by dividing the group difference in the change of least squares mean scores from baseline to posttreatment/follow-up by the pooled standard deviation for the compared groups at baseline. All analyses were conducted using SPSS version 26.

## Results

### Baseline Data and Participant Flow

**Figure 1.**
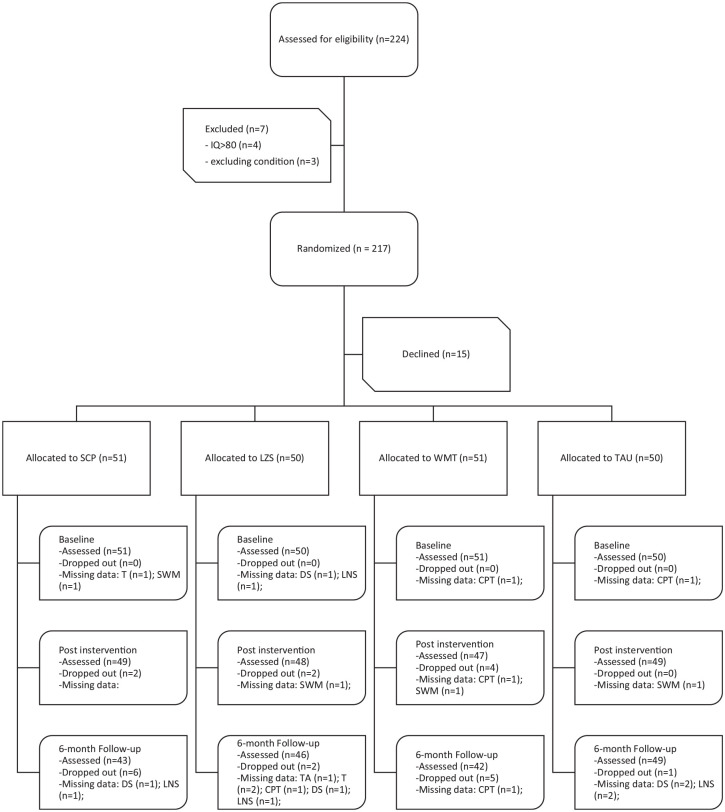
CONSORT flow diagram. *Note.* T = tapping; TA = time anticipation; SWM = spartial working memory task; CPT = continuous performance task; DS = digit span; LNS = letter-number-sequencing.

Two-hundred-twenty-four children and adolescents were assessed for the study. Four were excluded due to IQ scores below 80, while an additional three had conditions that were deemed to interfere with the study interventions. A total of 217 participants were included and randomized. Fifteen participants declined before the posttreatment assessment due to practical and logistical reasons, leaving *N* = 202. Drop-out was relatively low at posttreatment and follow-up (Figure 1).

Table 1 provides an overview of sample characteristics. The mean age was similar across groups (range: 12.2–12.6 years). The male to female ratio was about 3:1 in the NF groups and TAU, and 4:1 in WMT. There were no meaningful group differences in IQ, ADHD severity, or comorbidity. The ratio of ADHD presentations (combined/predominantly hyperactive vs. predominantly inattentive) varied somewhat between the groups, with a ratio of 3:2 in the NF groups, 4:1 in WMT, and close to 1:1 in TAU (WMT vs. TAU: *X*^2^[1, *N* = 101] = 6.748, *p* = .009). The use of medication was the lowest in the SCP group (49%), and the highest in the TAU group (70%), with a significant difference between these two groups (SCP vs. TAU: *X*^2^[1, *N* = 101] = 4.608, *p* = .032).

**Table 1.** Baseline Characteristics Per Intervention.

	Slow cortical potentials	Live *Z*-score	Working memory training	Treatments-as-usual
*N* = 202	51	50	51	50
Age in years, *M* (*SD*)	12.35 (2.65)	12.41 (2.30)	12.61 (2.74)	12.21 (2.41)
Male:Female	38:13	37:13	42:9	36:14
IQ, *M* (*SD*)	104.96 (15.35)	101.80 (12.74)	101.96 (15.87)	100.44 (14.80)
ADHD severity—teacher rating *t*-value, *M* (*SD*)	62.76 (13.37)	65.83 (14.88)	64.27 (15.47)	66.67 (14.61)
ADHD severity—parent rating *t*-value, *M* (*SD*)	80.51 (13.91)	82.84 (9.16)	81.32 (12.85)	82.84 (10.91)
ADHD severity—self rating *t*-value, *M* (*SD*)	69.24 (16.15)	67.12 (15.15)	72.26 (16.67)	70.06 (15.79)
ASD comorbid ASD, *n* (%)	8^a^ (16%)	7^b^ (14%)	7^c^ (14%)	12^d^ (24%)
Comorbid psychiatric disorder, *n* (%)	18 (35%)	15 (30%)	18 (35%)	17 (34%)
Predominantly inattentive presentation, *n* (%)	20 (39%)	19 (38%)	11^f^ (22%)	23^f^ (46%)
ADHD—medication, *n* (%)	25^g^ (49%)	32 (64%)	33 (65%)	35^g^ (70%)
Melatonin use, *n* (%)	6 (12%)	4 (8%)	3 (6%)	7 (14%)

a Childhood autism (CA) × 1, atypical autism (AA) × 2, asperger syndrome (AS) × 5.

b AA × 1, AS × 6.

c AA × 3, AS × 4.

d CA × 3, AA × 4, AS × 5.

e Include mood disorders, anxiety disorders, oppositional defiant disorder, sleeping disorders, learning disorders, and speech disorders;

f WMT versus TAU: *X*^2^(1, *N* = 101) = 6.748, *p* = .009.

g SCP versus TAU: *X*^2^(1, *N* = 101) = 4.608, *p* = .032.

### Immediate Effects

Compared to TAU, we found no effect of SCP or LZT at post-treatment on any measure (Table 2). However, WMT showed a significant effect on digit span forward (1.17; CI: 0.13–2.21; *p* = .028; *d*′ = 0.39); block tapping forward (1.69; CI: 0.95–2.43; *p* < .001; *d*′ = 0.79) and backwards (1.24; CI: 0.34–2.15; *p* = .008; *d*′ = 0.55); as well as on the ADHD-index score of the CPT-II (7.41; CI: 0.38–14.44; *p* = .039; *d*′ = 0.38). The groups’ mean scores and standard deviations for each time point are presented in the Supplemental Table S1a and S1b.

**Table 2.** Comparison Active Interventions to Treatment-as-Usual From Baseline to Posttreatment.

Measurement	Slow cortical potentials vs. treatment-as-usual			Live *Z*-score vs. treatment-as-usual			Working memory training vs. treatment-as-usual		
	Treatment effect (95% CI)	Sig.	Cohens *d′*	Treatment effect (95% CI)	Sig.	Cohens *d′*	Treatment effect (95% CI)	Sig.	Cohens *d*′
Digit span—forward^↑a^	−0.29 (−1.19 to 0.62)	0.529	−0.12	−0.31 (−1.21 to 0.60)	0.505	−0.12	**1.17 (0.13 to 2.21)**	**0.028**	**0.39**
Digit span- backward^↑a^	−0.38 (−1.53 to 0.77)	0.513	−0.14	−0.59 (−1.58 to 0.41)	0.245	−0.21	0.49 (−0.72 to 1.70)	0.426	0.18
Number letter sequences^↑a^	−0.73 (−1.68 to 0.22)	0.129	−0.26	−0.77 (−1.64 to 0.09)	0.079	−0.27	−0.32 (−1.23 to 0.59)	0.491	−0.11
Block tapping—forward^↑b^	−0.25 (−0.96 to 0.47)	0.498	−0.11	−0.26 (−0.96 to 0.43)	0.455	−0.12	**1.69 (0.95 to 2.43)**	<**0.001**	**0.79**
Block tapping—backward^↑b^	0.09 (−0.77 to 0.94)	0.841	0.04	−0.14 (−1.07 to 0.80)	0.773	−0.06	**1.24 (0.34 to 2.15)**	**0.008**	**0.55**
Telephone task—BSE^↓^	−1.36 (−5.15 to 2.44)	0.480	−0.14	0.16 (−4.14 to 4.47)	0.940	0.02	−2.26 (−6.29 to 1.78)	0.270	−0.23
Telephone task—WSE^↓^	−0.70 (−1.89 to 0.48)	0.242	−0.40	−0.76 (−1.99 to 0.48)	0.229	−0.40	−1.18 (−2.44 to 0.07)	0.065	−0.56
CPT-II—omissions^↓^	0.53 (−3.63 to 4.68)	0.802	0.05	1.61 (−2.61 to 5.84)	0.451	0.16	4.13 (−0.51 to 8.77)	0.081	0.35
CPT-II—commissions^↓^	−0.75 (−3.78 to 2.27)	0.623	−0.08	1.53 (−1.43 to 4.48)	0.309	0.14	2.19 (−0.98 to 5.35)	0.174	0.22
CPT-II—hit RT^↓^	1.13 (−2.11 to 4.37)	0.490	0.10	0.40 (−2.72 to 3.53)	0.799	0.04	1.50 (−1.67 to 4.67)	0.350	0.13
CPT-II—hit RT SE^↓^	0.61 (−2.40 to 3.62)	0.690	0.06	0.17 (−3.23 to 3.57)	0.920	0.02	3.30 (−0.32 to 6.92)	0.074	0.35
CPT-II—ADHD-index^↓c^	1.82 (−4.61 to 8.25)	0.576	0.10	3.20 (−2.98 to 9.38)	0.307	0.18	**7.41 (0.38 to 14.44)**	**0.039**	**0.38**
Tapping—CoV^↓^	2.21 (−0.76 to 5.17)	0.143	0.29	1.23 (−1.53 to 3.99)	0.380	0.16	−1.09 (−5.19 to 3.02)	0.601	−0.09
TA 400 ms—hit rate ^↑^	0.01 (−0.06 to 0.07)	0.864	0.04	0.00 (−0.06 to 0.05)	0.892	−0.03	0.01 (−0.06 to 0.07)	0.808	0.05
TA 400 ms—too early^↓^	−0.01 (−0.06 to 0.04)	0.634	−0.10	0.01 (−0.04 to 0.06)	0.767	0.06	0.00 (−0.05 to 0.05)	0.947	0.01
TA 2,000 ms—hit rate^↑^	−0.04 (−0.13 to 0.05)	0.422	−0.14	−0.09 (−0.18 to 0.00)	0.058	−0.35	0.00 (−0.09 to 0.10)	0.922	0.02
TA 2,000 ms—too early^↓^	−0.01 (−0.10 to 0.08)	0.825	−0.04	0.08 (−0.01 to 0.16)	0.098	0.32	−0.01 (−0.11 to 0.08)	0.768	−0.05

*Note.* Significant results are bold. ^↓^ = negative values favor the first intervention; ^↑^ = positive values favor the first intervention; BSE = between-search-errors (raw score); WSE = within-search-errors (raw score); CPT-II = Conners’ continuous performance task (*t*-scores; 50 ± 10); CoV = coefficient of variability (*SD*/mean tapping rate × 100); TA = time anticipation (percentages; max score 1.00); RT = reaction time; *SE* = standard error.

a Scale scores (10 ± 3).

b Raw scores (max score 14).

c Percentages (max score 100).

When comparing the active interventions against each other, WMT was superior to both SCP and LZT on digit span forward and on both block-tapping modalities. Furthermore, the WMT group had more correct responses on the 2000 ms. TA task than LZT (0.09; CI: 0.01–0.18; *p* = .034; *d*′ = −0.39), while too early responses were more common in LZT than WMT (−0.09; CI: −0.17 to −0.01; *p* = .037; *d*′ = −0.39). Similarly, too early responses were more common for LZT than SCP (0.09; CI: 0.00–0.17; *p* = .040; *d*′ = 0.37). Posttreatment comparisons between the active interventions are presented in Supplemental Table S2.

### Sustained Effects

At follow-up, there were no significant differences between SCP and TAU (Table 3). However, there was a significant difference favoring TAU compared to LZT on digit span forward (−0.98; CI: −1.90 to −0.07; *p* = .035; *d*′ = −0.40). For WMT there were significant effects only for block tapping (forward: 1.26; CI: 0.48–2.05; *p* = .002; *d*′ = 0.59; backward: 1.31; CI: 0.38–2.23; *p* = 0.006; *d*′ = 0.57). When comparing the active interventions, the differences between SCP and WMT on both block tapping modalities remained (forward: 1.55; CI: 0.80–2.30; *p* < .001; *d*′ = 0.77; backward: 0.93; CI. 0.03–1.82; *p* = .043; *d*′ = 0.41). WMT was also superior to LZT on digit span forward (1.53; CI: 0.50–2.55; *p* = .004; *d*′ = 0.49) and block-tapping forward (1.90; 1.07–2.73; *p* < .001; *d*′ = 0.96). Also, there was a difference between LZT and WMT for the coefficient of variation of the Tapping task (−2.86; CI: −5.60 to −0.12; *p* = .041; *d*′ = −0.26), and on correct responses on the 2,000 ms. TA task (0.11; CI: 0.01–0.20; *p* = .024; *d*′ = 0.44). Compared to LZT, the SCP group had significant higher score on digit span forward (1.12; CI: 0.19–2.05; *p* = .019; *d*′ = 0.44). Follow-up comparisons between the active interventions are presented in Supplemental Table S3.

**Table 3.** Comparison Active Interventions to Treatment-as-Usual from Baseline to 6-Month Follow-Up.

Measurement	Slow cortical potentials vs. treatment-as-usual			Live *Z*-score vs. treatment-as-usual			Working memory training vs. treatment-as-usual		
	Treatment effect (95% CI)	Sig.	Cohens *d*′	Treatment effect (95% CI)	Sig.	Cohens *d*′	Treatment effect (95% CI)	Sig.	Cohens *d*′
Digit span—forward^↑a^	0.11 (−0.78 to 1.00)	0.803	0.05	−**0.98 (**−**1.90 to** −**0.07)**	**0.035**	−**0.40**	0.55 (−0.43 to 1.53)	0.267	0.18
Digit span—backward^↑a^	−0.98 (−2.18 to 0.22)	0.107	−0.35	−0.40 (−1.55 to 0.74)	0.485	−0.14	0.20 (−0.96 to 1.36)	0.737	0.07
Number letter sequences^↑a^	−0.92 (−2.05 to 0.20)	0.108	−0.33	−0.57 (−1.59 to 0.46)	0.276	−0.20	−0.11 (−1.28 to 1.05)	0.848	−0.04
Block tapping—forward^↑b^	−0.34 (−1.09 to 0.41)	0.374	−0.16	−0.66 (−1.49 to 0.17)	0.118	−0.31	**1.26 (0.48 to 2.05)**	**0.002**	**0.59**
Block tapping—backward^↑b^	0.34 (−0.54 to 1.23)	0.444	0.16	0.36 (−0.57 to 1.29)	0.445	0.17	**1.31 (0.38 to 2.23)**	**0.006**	**0.57**
Telephone task—BSE^↓^	2.73 (−0.94 to 6.39)	0.143	0.27	2.40 (−1.67 to 6.46)	0.245	0.22	2.37 (−1.49 to 6.24)	0.227	0.25
Telephone task—WSE^↓^	−0.03 (−0.89 to 0.83)	0.951	−0.02	−0.46 (−1.38 to 0.46)	0.322	−0.25	−0.53 (−1.49 to 0.43)	0.279	−0.25
CPT-II—omissions^↓^	−2.39 (−7.23 to 2.44)	0.329	−0.22	−3.41 (−7.86 to 1.05)	0.132	−0.35	−1.28 (−6.53 to 3.97)	0.630	−0.11
CPT-II—commissions^↓^	0.37 (−3.09 to 3.83)	0.832	0.04	−1.84 (−5.36 to 1.67)	0.300	−0.17	−0.08 (−3.78 to 3.63)	0.968	−0.01
CPT-II—hit RT^↓^	0.54 (−2.70 to 3.77)	0.744	0.05	0.73 (−2.43 to 3.89)	0.648	0.07	1.70 (−1.74 to 5.14)	0.329	0.15
CPT-II—hit RT SE^↓^	−0.50 (−3.93 to 2.92)	0.772	−0.05	−2.08 (−5.73 to 1.57)	0.262	−0.21	−0.76 (−4.32 to 2.81)	0.675	−0.08
CPT-II—ADHD-index^↓c^	0.29 (−6.59 to 7.18)	0.933	0.02	−1.10 (−7.59 to 5.38)	0.737	−0.06	1.32 (−6.27 to 8.91)	0.731	0.07
Tapping—CoV^↓^	1.01 (−2.55 to 4.58)	0.573	0.13	0.39 (−2.68 to 3.45)	0.802	0.05	−2.45 (−5.71 to 0.80)	0.138	−0.20
TA 400 ms—Hit rate ^↑^	−0.02 (−0.08 to 0.05)	0.647	−0.10	−0.03 (−0.09 to 0.03)	0.321	−0.21	0.01 (−0.05 to 0.08)	0.703	0.08
TA 400 ms—Too early^↓^	0.00 (−0.06 to 0.06)	0.982	0.01	0.01 (−0.04 to 0.07)	0.588	0.12	−0.03 (−0.08 to 0.03)	0.321	−0.24
TA 2,000 ms—Hit rate^↑^	−0.02 (−0.12 to 0.07)	0.652	−0.08	−0.08 (−0.16 to 0.00)	0.058	−0.33	0.02 (−0.07 to 0.12)	0.639	0.09
TA 2,000 ms—Too early^↓^	−0.02 (−0.12 to 0.07)	0.668	−0.08	0.05 (−0.03 to 0.13)	0.217	0.22	−0.03 (−0.12 to 0.06)	0.518	−0.12

*Note.* Significant results are bold. ^↓^ = negative values favor the first intervention; ^↑^ = positive values favor the first intervention; BSE = between-search-errors (raw score); WSE = within-search-errors (raw score); CPT-II = Conners’ continuous performance task (*t*-scores; 50 ± 10); CoV = coefficient of variability (*SD*/mean tapping rate* ×* 100); TA = time anticipation (percentages; max score 1.00); RT = reaction time; *SE* = Standard Error.

a Scale scores (10 ± 3).

b Raw scores (max score 14).

c Percentages (max score 100).

### Sensitivity Analyses

Age was moderating the immediate effects observed between WMT and TAU for digit span forward (2.56; CI: 0.38–4.75; *p* = .022). Stratified analyses revealed that benefits were more pronounced for the adolescents (2.78; CI: 0.54–5.02; *p* = .016) than for the children (0.19; CI: −0.91 to 1.28; *p* = .74). Similarly, the effect of WMT compared to SCP on digit span was also moderated by age (3.01; CI: 0.88–5.14; *p* = .006), with a significant effect for adolescents (3.35; CI: 1.23–5.48; *p* = .003) but not for children (0.30; CI: −0.78 to 1.38; *p* = .58). Age was also moderating the sustained effect between SCP and WMT for block tapping forward (1.82; CI: 0.21–3.42; *p* = .027), with greater effects for the adolescents (2.84; CI: 0.15–1.85; *p* = .001) than for the children (1.00; CI: 0.15 to 1.85; p = 0.022). None of the other observed effects were significantly moderated by age or sex.

Baseline imbalance was indicated for ADHD presentation and ADHD medication. Consequently, the moderating effect of these two variables were explored for all outcomes, A moderating effects of the ADHD presentations at posttreatment were for only detected for LZT compared to TAU, regarding the hit RT on the CPT-II (−6.83; CI: −13.13 to −0.52; *p* = .034) and block tapping forwards (1.54; CI: 0.15–2.93; *p* = .030). Stratified analyses suggested that LZT had more beneficial effects for the predominantly inattentive presentation. At follow-up, an interaction for block tapping forward for LZT compared to TAU remained (2.28; CI: 0.62–3.94; *p* = .007), favoring the predominantly inattentive presentation. The follow-up analysis also found a moderating effect of ADHD presentations for SCP compared to TAU on digit span forward (−2.36; CI: −4.11 to −0.61; *p* = .009), indicating more benefits for the combined subtype. Stratified baseline characteristics (Supplemental Table S4a and S4b) and results for the ADHD presentations at posttreatment (Supplemental Table S5a and S5b) and follow-up (Supplemental Table S6a and S6b) are available in the Supplemental Material.

We found a significant interaction of time by group by medication status for omission errors at posttreatment, for both SCP (15.59; CI: 7.44–23.74; *p* < .001) and LZT (10.59; CI: 1.95–19.23; *p* = .017) compared to TAU. Stratified analyses suggested that medicated participants performed better in the SCP group, while none-medicated participants performed worse compared to TAU. Further interactions at posttreatment were found for SCP compared to TAU for the CPT-II ADHD-index (14.19; CI: 0.96–27.41; *p* = .036), indicating favorable results for the medicated participants, and the Coefficient of variability of the Tapping task (7.41; CI: 1.30–13.52; *p* = .018), indicating less variability of the non-medicated participant. No significant interactions were found at follow-up. Results of the stratified analysis based on medication status is presented in the Supplemental Table S7a, S7b, S8a, and S8b. Finally, comparisons between learners and non-learners within the SCP group did not reveal any significant differences in outcome.

## Discussion

This study examined the effect of the neurocognitive training methods on working memory, time perception, and attention/inhibition functions in children and adolescents with ADHD. Both immediate and sustained effects (6-month follow-up) were assessed, as well as the moderating role of clinical ADHD presentations. To increase the clinical relevance of the findings, the study utilized commercially available equipment/software, participants with common comorbidities, and cognitive tests that are common in clinical practice. Furthermore, staff were to a large extent research assistants, trained inhouse. A similar level of training seems likely if NF would be implementing broadly in public outpatient healthcare services or a school-setting. No benefits of Slow Cortical Potential (SCP) or Live *Z*-Score training (LZT) over Treatment-as-usual (TAU) were observed on any of the targeted outcomes. Working memory training (WMT) showed improvements compared to TAU and neurofeedback (NF) on some working-memory tasks, but only the effects on the block tapping tasks (forward and backwards) were sustained at follow-up. Overall, we found no clear indication that effects were moderated by the different ADHD presentations. However, there were some tendencies for greater benefits for adolescents compared to the younger participants, concerning some memory measures.

WMT showed effects on some working memory tasks (i.e., block tapping and digit span forward), but not on number-letter-sequencing or the “telephone task”. This is in line with previous findings on immediate and sustained effects for visuospatial and verbal working-memory (Melby-Lervåg et al., 2016). Incidentally, the WMT program we examined, Minneslek flex™, includes games that are similar to both digit span and block tapping tasks, but not the other working memory outcomes. This suggests that the effects may not generalize across the full range of working memory functions, supporting previous observations that WMT mainly has near-transfer effects (Melby-Lervåg et al., 2016).

While no significant effects on any inhibition nor attention measures (i.e., CPT-II tasks) were observed, we did find a significant improvement for WMT over TAU on the ADHD-index combining multiple measures of the CPT-II. This indicates that the training has small and inconsistent effects on the individual cognitive functions, which nevertheless may sum up to detectable effects on composite measures. The lack of significant effects of SCP or LZT was unexpected. In fact, the group receiving LZT declined in performance on digit span forward at follow-up. However, this most likely reflects measurement errors or motivational factors, rather than actual change in functioning. Previous research has suggested improvement from NF on inhibition tasks of moderate effect sizes, based on different measures of continuous performance tasks (Lambez et al., 2020).

It should be noted that the effects of WMT were observed exclusively on non-executive memory (i.e., simple recall, such as in the tasks’ forward modalities). Improvements on block tapping also included the backwards modality, but the task was administered face-to-face and there was no forced delay between stimuli and response, as would have been the case in a computer-based ditto. Instead, it may be argued that also the backwards modality mainly relied on non-executive memory, as the need for manipulating the stimuli was limited. The lack of far transfer effects suggests that the effects of WMT may be due to improvements in the application of strategies rather than general and transferable changes in the working memory capacities.

The negative findings reported for many outcomes in this study should be interpreted with caution. Considering the well documented heterogeneity of cognitive alterations in ADHD (Pievsky & McGrath, 2018), it is quite possible that clinically relevant improvements are limited to a subsample of children and adolescents with ADHD. Future research should continue exploring how different neurocognitive methods affect cognitive functions, as this could facilitate both improved matching and personalization. Equally important, improvement of neurocognitive testing and analytical methods are needed to detect change in clinical characteristics and subtle cognitive differences (Ging-Jehli et al., 2021).

We did not find differences in outcome between the participants in the SCP group who self-regulated successfully, and those who did not. However, future research should continue to address what role performance plays during neurocognitive interventions, as there are indications that compliance and motivation may effect perceived outcome (Hasslinger et al., 2020). Further inquiries into how ADHD patients with different cognitive profiles respond to different interventions is also warranted, including neurocognitive training methods that have not been addressed in this study. Sub-analyses for both the interaction with age and the interaction with sex indicated that many significant results were influenced by changes in TAU rather than differences in the intervention group. Overall, no clear patterns were found for age nor sex differences.

Our findings must also be interpreted with some limitations in mind. First, the staff that conducted the training also administered the assessments. Based on daily interaction, the relationship between trainer and subject tended to become more casual, especially since helping the participant to maintain motivation is important for adherence. While this may have increased the level of comfort during a stressful testing situation, it may also in some cases have led to an overly relaxed atmosphere, preventing maximal performance. Subjects in the TAU group, on the other hand, only met the staff in their role as assessors. Tasks administered face-to-face in particular were susceptible to such risk of bias. Fully computerized tests would potentially decrease such a risk. Second, participants that were medicated for ADHD, had to undergo a 48-hour washout period prior the assessments, which may have had a negative impact on some participants performance and increased measurement errors. Third, the sample size of each group was modest, especially when considering the heterogeneity of the target population. Factors such as age, sex, the ADHD sub-type, ASD comorbidity, medication, and symptom severity all contribute toward the diversity of the sample. Fourth, there were some baseline differences, particularly regarding ADHD presentation and ADHD-medication status. However, our moderator analyses and stratified analysis did not suggest that these factors had substantial impacts on the outcome. Nonetheless, stratified analysis may suggest that for SCP the 48-h washout period prior each assessment did not have the same effect on performance on CPT-II omission errors and its ADHD-index. However, the lack of significant effects at follow-up negates this. Fifth, implementing standard sites in LZT instead of individual sites may have limit the outcome. Sixth, although we implemented cards to facilitate the transfer of neural self-regulation into everyday life, we do not know to what extent the transfer was successful. More supporting strategies concerning generalizability may be needed.

In conclusion, we found no support for broad effects of NF on multiple cognitive functions associated in ADHD. More inquiries into individual effects are needed in order to enable more personalized treatment options. In particular, future research should focus more on how neurocognitive training can be optimized for children and adolescents with diverse cognitive profiles.

## Supplemental Material

sj-docx-1-jad-10.1177_10870547211063645 – Supplemental material for Immediate and Sustained Effects of Neurofeedback and Working Memory Training on Cognitive Functions in Children and Adolescents with ADHD: A Multi-Arm Pragmatic Randomized Controlled Trial Click here for additional data file. Supplemental material, sj-docx-1-jad-10.1177_10870547211063645 for Immediate and Sustained Effects of Neurofeedback and Working Memory Training on Cognitive Functions in Children and Adolescents with ADHD: A Multi-Arm Pragmatic Randomized Controlled Trial by John Hasslinger, Ulf Jonsson and Sven Bölte in Journal of Attention Disorders
