# Supplementary material for: Immediate and Sustained Effects of Neurofeedback and Working Memory Training on Cognitive Functions in Children and Adolescents with ADHD: A Multi-Arm Pragmatic Randomized Controlled Trial
Source: J Atten Disord. 2022 Jan 16;26(11):1492–506. doi: 10.1177/10870547211063645 (PMC9277335; doi:10.1177/10870547211063645)
Supplement: sj-docx-1-jad-10.1177_10870547211063645 – Supplemental material for Immediate and Sustained Effects of Neurofeedback and Working Memory Training on Cognitive Functions in Children and Adolescents with ADHD: A Multi-Arm Pragmatic Randomized Controlled Trial [file sj-docx-1-jad-10.1177_10870547211063645.docx]

**Immediate and Sustained Effects of Neurofeedback and Working Memory Training on Cognitive Functions in Children and Adolescents with ADHD: A Multi-Arm Pragmatic Randomized Controlled Trial**

John Hasslinger^1,2, *^, Ulf Jonsson^1,2,3^ & Sven Bölte^1,2,4^

¹Center of Neurodevelopmental Disorders (KIND), Centre for Psychiatry Research; Department of Women’s and Children’s Health, Karolinska Institutet & Child and Adolescent Psychiatry, Stockholm Health Care Services, Region Stockholm, Stockholm, Sweden.

^2^Child and Adolescent Psychiatry, Stockholm Health Services, Stockholm, Sweden.

^3^Department of Neuroscience, Child and Adolescent Psychiatry, Uppsala University, Uppsala, Sweden.

^4^Curtin Autism Research Group, Curtin School of Allied Health, Curtin University, Perth, Western Australia.

*john.hasslinger@ki.se

|  |  |
| --- | --- |
| **Supplement Table S1a**: Mean raw scores for CPT-II tasks for complete cases per intervention, at baseline, posttreatment and follow-up. | Page 2 |
| **Supplement Table S1b**: Mean scores for complete cases per intervention, at baseline, posttreatment  and follow-up. | Page 3 |
| **Supplement Table S2**: Results from comparison of the active interventions from baseline to posttreatment. | Page 4 |
| **Supplement Table S3**: Results from comparison of the active interventions from baseline to 6-month follow-up. | Page 5 |
| **Supplement Table S4a**: Baseline characteristics of participants with ADHD combined presentation. | Page 6 |
| **Supplement Table S4b**: Baseline characteristics of participants with ADHD predominantly inattentive presentation. | Page 7 |
| **Supplement Table S5a:** Results for active interventions compared to treatment-as-usual from baseline to posttreatment, for participants with ADHD combined presentation. | Page 8 |
| **Supplement Table S5b:** Results for active interventions compared to treatment-as-usual from baseline to posttreatment, for participants with ADHD predominantly inattentive presentation. | Page 9 |
| **Supplement Table S6a:** Results for active interventions compared to treatment-as-usual from baseline to 6-month follow-up, for participants with ADHD combined presentation. | Page 10 |
| **Supplement Table S6b:** Results for active interventions compared to treatment-as-usual from baseline to 6-month follow-up, for participants with ADHD predominantly inattentive presentation. | Page 11 |
| **Supplement Table S7a** Results for active interventions compared to treatment-as-usual from baseline to posttreatment, for medicated-participants. | Page 12 |
| **Supplement Table S7b** Results for active interventions compared to treatment-as-usual from baseline to posttreatment, for non-medicated participants. | Page 13 |
| **Supplement Table S8a** Results for active interventions compared to treatment-as-usual from baseline to 6-month follow-up, for medicated-participants. | Page 14 |
| **Supplement Table S8b** Results for active interventions compared to treatment-as-usual from baseline to 6-month follow-up, for non-medicated participants. | Page 15 |
|  |  |

| **Supplement Table S1a** Mean raw scores for CPT-II, for complete cases per intervention, at baseline, posttreatment and follow-up | | | | | | | | | |  |  |
| --- | --- | --- | --- | --- | --- | --- | --- | --- | --- | --- | --- |
|  |  | **Slow Cortical**  **Potential** | | **Live Z-Score** | | **Working Memory**  **Training** | | **Treatment as Usual** | |  |  |
| **Measure** | **Time** | **Mean (SD)** | **n** | **Mean (SD)** | **n** | **Mean (SD)** | **n** | **Mean (SD)** | **n** |  |  |
| CPT-II – Omissions | Baseline | 16.37  (15.82) | 51 | 15.44  (13.37) | 50 | 18.70  (20.24) | 50 | 15.76  (16.24) | 49 |  |  |
|  | Post | 17.59  (13.28) | 49 | 20.06  (24.64) | 48 | 23.59  (23.18) | 46 | 17.43  (20.05) | 49 |  |  |
|  | FU | 15.28  (14.25) | 43 | 14.02  (14.69) | 45 | 18.22 (  18.94) | 41 | 15.92  (16.20) | 49 |  |  |
| CPT-II –Commissions^a^ | Baseline | 22.12  (5.88) | 51 | 22.08  (7.28) | 50 | 21.26  (7.19) | 50 | 21.47  (6.70) | 49 |  |  |
|  | Post | 19.55  (7.69) | 49 | 21.27  (7.73) | 48 | 20.80  (7.66) | 46 | 19.80  (6.72) | 49 |  |  |
|  | FU | 21.09  (8.21) | 43 | 20.13  (7.32) | 45 | 19.93  (7.90) | 41 | 20.73  (8.08) | 49 |  |  |
| CPT-II –  Hit RT | Baseline | 444.47 (81.63) | 51 | 433.28 (76.62) | 50 | 439.82  (93.04) | 50 | 449.77  (99.75) | 49 |  |  |
|  | Post | 458.09 (87.32) | 49 | 444.95 (95.29) | 48 | 457.99 (109.23) | 46 | 456.28  (97.37) | 49 |  |  |
|  | FU | 442.84 (87.14) | 43 | 429.35 (80.57) | 45 | 442.92  (95.54) | 41 | 439.07  (90.07) | 49 |  |  |
| CPT-II –  Hit RT SE | Baseline | 12.79  (7.82) | 51 | 11.87  (6.77) | 50 | 12.11  (6.94) | 50 | 12.53  (7.27) | 49 |  |  |
|  | Post | 12.56  (6.55) | 49 | 12.67  (8.08) | 48 | 14.14  (8.82) | 46 | 13.18  (8.81) | 49 |  |  |
|  | FU | 12.53  (7.74) | 43 | 11.02  (6.41) | 45 | 11.54  (6.75) | 41 | 12.10  (6.94) | 49 |  |  |
| CPT-II –Commission RT | Baseline | 408.88 (114.31) | 51 | 397.15 (87.58) | 50 | 398.82  (87.53) | 50 | 403.69  (96.96) | 49 |  |  |
|  | Post | 420.47 (139.36) | 49 | 383.06 (75.79) | 47 | 409.30 (140.09) | 45 | 397.91  (101.38) | 48 |  |  |
|  | FU | 398.35 (106.32) | 42 | 385.60 (94.04) | 44 | 389.39 (113.00) | 41 | 400.81  (122.27) | 48 |  |  |
|  |  |  |  |  |  |  |  |  |  |  |  |
| **Note**. ^a^Maximum number of Commissions = 36; SD = Standard Deviation; RT = Reaction Time; SE = Standard Error; Post = Posttreatment; FU = 6-month Follow-up; n = Number of completed cases.  Discrepancy of *n* at Commission RT, due to participants that did not commit any commission errors. | | | | | | | | | |  |  |

| **Supplement Table S1b** Mean scores for complete cases per intervention, at baseline, posttreatment  and follow-up | | | | | | | | | |
| --- | --- | --- | --- | --- | --- | --- | --- | --- | --- |
|  |  | **Slow Cortical**  **Potential** | | **Live Z-Score** | | **Working Memory**  **Training** | | **Treatment as Usual** | |
| **Measure** | **Time** | **Mean (SD)** | **n** | **Mean (SD)** | **n** | **Mean (SD)** | **n** | **Mean (SD)** | **n** |
| Digit Span –  forward^↑^ | Baseline | 7.82 (2.44) | 51 | 7.45 (2.60) | 49 | 7.78 (3.53) | 51 | 7.06 (2.32) | 50 |
|  | Post | 7.86 (3.16) | 49 | 7.54 (3.20) | 48 | 9.43 (3.74) | 47 | 7.39 (2.61) | 49 |
|  | FU | 8.21 (2.88) | 42 | 7.04 (3.19) | 45 | 8.86 (3.73) | 42 | 7.52 (2.67) | 48 |
| Digit Span –  backward^↑^ | Baseline | 9.47 (3.04) | 51 | 8.47 (3.10) | 49 | 8.84 (2.82) | 51 | 9.10 (2.55) | 50 |
|  | Post | 9.55 (3.25) | 49 | 8.42 (2.89) | 48 | 9.89 (3.58) | 47 | 9.57 (2.72) | 49 |
|  | FU | 9.19 (2.73) | 42 | 8.93 (2.87) | 45 | 9.90 (3.05) | 42 | 9.81 (2.97) | 48 |
| Number Letter Sequences^↑^ | Baseline | 9.22 (2.55) | 51 | 8.24 (2.59) | 49 | 8.76 (2.57) | 51 | 8.20 (3.02) | 50 |
|  | Post | 9.33 (2.13) | 49 | 8.29 (2.57) | 48 | 9.36 (2.59) | 47 | 9.02 (2.31) | 49 |
|  | FU | 9.38 (2.17) | 42 | 8.78 (2.26) | 45 | 9.86 (3.19) | 42 | 9.29 (2.63) | 48 |
| Block Tapping –  forward^↑^ | Baseline | 8.22 (2.03) | 51 | 7.80 (1.96) | 50 | 7.67 (2.00) | 51 | 7.58 (2.30) | 50 |
|  | Post | 8.20 (2.27) | 49 | 7.81 (1.85) | 48 | 9.66 (2.08) | 47 | 7.86 (1.93) | 50 |
|  | FU | 8.38 (2.02) | 42 | 7.64 (1.91) | 45 | 9.44 (1.99) | 41 | 8.13 (2.11) | 48 |
| Block Tapping – backward^↑^ | Baseline | 7.41 (2.11) | 51 | 6.90 (2.17) | 50 | 6.75 (2.39) | 51 | 7.10 (2.16) | 50 |
|  | Post | 7.80 (2.00) | 49 | 7.13 (1.84) | 48 | 8.36 (2.49) | 47 | 7.44 (2.27) | 50 |
|  | FU | 7.83 (2.27) | 42 | 7.40 (1.72) | 45 | 8.17 (2.57) | 41 | 7.25 (2.26) | 48 |
| Telephone task –  BSE**^↓^** | Baseline | 9.78 (9.00) | 50 | 11.50 (10.65) | 50 | 9.59 (8.46) | 51 | 12.10 (10.79) | 50 |
|  | Post | 8.21 (8.59) | 48 | 11.34 (9.72) | 47 | 6.87 (8.12) | 46 | 11.77 (11.13) | 48 |
|  | FU | 8.26 (8.30) | 43 | 9.57 (10.08) | 44 | 7.31 (8.34) | 42 | 7.73 (8.30) | 49 |
| Telephone task –  WSE**^↓^** | Baseline | 1.04 (1.24) | 50 | 1.36 (1.52) | 50 | 1.47 (2.08) | 51 | 1.28 (2.17) | 50 |
|  | Post | 0.73 (1.50) | 48 | 0.98 (1.44) | 47 | 0.67 (1.19) | 46 | 1.67 (3.49) | 48 |
|  | FU | 0.86 (1.23) | 43 | 0.73 (1.34) | 44 | 0.79 (1.32) | 42 | 1.12 (1.78) | 49 |
| CPT-II –  Omissions**^↓^** | Baseline | 52.97 (11.07) | 51 | 52.03 (8.81) | 50 | 53.78 (12.96) | 50 | 52.08 (10.70) | 49 |
|  | Post | 54.88 (13.45) | 49 | 55.31 (16.72) | 48 | 59.06 (17.46) | 46 | 53.32 (13.36) | 49 |
|  | FU | 51.76 (9.74) | 43 | 51.05 (9.75) | 45 | 54.54 (13.07) | 41 | 53.89 (15.16) | 49 |
| CPT-II –  Commissions**^↓^** | Baseline | 48.90 (9.42) | 51 | 48.89 (11.67) | 50 | 47.67 (10.63) | 50 | 47.98 (9.49) | 49 |
|  | Post | 45.15 (12.21) | 49 | 48.01 (12.43) | 48 | 47.03 (12.36) | 46 | 45.54 (9.51) | 49 |
|  | FU | 47.80 (11.52) | 43 | 46.33 (11.17) | 45 | 46.50 (12.33) | 41 | 47.68 (12.41) | 49 |
| CPT-II –  Hit RT**^↓^** | Baseline | 55.18 (10.29) | 51 | 54.23 (9.71) | 50 | 55.20 (10.73) | 50 | 55.86 (11.55) | 49 |
|  | Post | 57.47 (11.15) | 49 | 55.49 (12.10) | 48 | 57.70 (12.29) | 46 | 56.92 (11.48) | 49 |
|  | FU | 55.09 (11.61) | 43 | 54.29 (10.24) | 45 | 55.74 (10.89) | 41 | 55.04 (10.98) | 49 |
| CPT-II –  Hit RT SE**^↓^** | Baseline | 53.51 (9.07) | 51 | 52.46 (9.73) | 50 | 52.86 (8.83) | 50 | 53.39 (9.87) | 49 |
|  | Post | 54.56 (8.86) | 49 | 53.01 (10.47) | 48 | 56.45 (11.52) | 46 | 53.83 (11.04) | 49 |
|  | FU | 53.23 (9.91) | 43 | 51.12 (9.97) | 45 | 52.31 (9.15) | 41 | 54.05 (10.75) | 49 |
| CPT-II –  ADHD-index**^↓^** | Baseline | 54.84 (16.96) | 51 | 53.54 (15.63) | 50 | 56.25 (19.30) | 50 | 55.81 (20.14) | 49 |
|  | Post | 57.48 (17.65) | 49 | 57.45 (20.80) | 48 | 64.16 (20.15) | 46 | 56.53 (20.85) | 49 |
|  | FU | 54.62 (19.11) | 43 | 52.97 (19.98) | 45 | 56.51 (19.94) | 41 | 55.62 (19.59) | 49 |
| Tapping –  CoV**^↓^** | Baseline | 12.88 (6.56) | 50 | 11.18 (6.20) | 50 | 15.59 (15.16) | 51 | 12.92 (8.63) | 50 |
|  | Post | 14.40 (8.88) | 49 | 11.79 (6.05) | 48 | 13.55 (12.86) | 47 | 12.23 (6.90) | 49 |
|  | FU | 13.83 (10.78) | 43 | 11.42 (5.95) | 44 | 12.86 (11.88) | 42 | 12.50 (11.60) | 49 |
| TA 400ms –  Hit rate^↑^ | Baseline | 0.46 (0.28) | 51 | 0.54 (0.22) | 50 | 0.50 (0.26) | 50 | 0.49 (0.27) | 49 |
|  | Post | 0.51 (0.24) | 49 | 0.54 (0.25) | 48 | 0.60 (0.24) | 47 | 0.58 (0.23) | 48 |
|  | FU | 0.55 (0.27) | 43 | 0.58 (0.22) | 45 | 0.63 (0.26) | 42 | 0.61 (0.22) | 49 |
| TA 400ms –  Too Early**^↓^** | Baseline | 0.46 (0.26) | 51 | 0.37 (0.21) | 50 | 0.42 (0.24) | 50 | 0.42 (0.27) | 49 |
|  | Post | 0.39 (0.25) | 49 | 0.38 (0.24) | 48 | 0.34 (0.23) | 47 | 0.35 (0.24) | 48 |
|  | FU | 0.36 (0.25) | 43 | 0.33 (0.20) | 45 | 0.31 (0.23) | 42 | 0.32 (0.20) | 49 |
| TA 2000ms –  Hit rate^↑^ | Baseline | 0.75 (0.15) | 51 | 0.80 (0.14) | 50 | 0.74 (0.17) | 51 | 0.76 (0.15) | 50 |
|  | Post | 0.82 (0.14) | 49 | 0.87 (0.12) | 48 | 0.82 (0.15) | 47 | 0.83 (0.13) | 49 |
|  | FU | 0.83 (0.13) | 43 | 0.86 (0.11) | 45 | 0.84 (0.14) | 42 | 0.85 (0.13) | 48 |
| TA 2000ms –  Too Early**^↓^** | Baseline | 0.18 (0.13) | 51 | 0.15 (0.11) | 50 | 0.19 (0.12) | 51 | 0.18 (0.12) | 50 |
|  | Post | 0.12 (0.11) | 49 | 0.10 (0.10) | 48 | 0.13 (0.11) | 47 | 0.13 (0.10) | 49 |
|  | FU | 0.12 (0.12) | 43 | 0.10 (0.09) | 45 | 0.10 (0.11) | 42 | 0.12 (0.11) | 48 |
| **Note**. **^↓^**= lower scores are sought; ^↑^**=** higher scores are sought; BSE = Between-search-errors; WSE = Within-search-errors; CPT-II = Conners’ Continuous Performance task; CoV = Coefficient of Variability; TA = Time Anticipation; RT = Reaction Time; SE = Standard Error Post = Posttreatment; FU = 6-month Follow-up; n = Number of completed cases | | | | | | | | | |

| **Supplement Table S2** Comparison between the active interventions from baseline to posttreatment | | | | | | | | | | |
| --- | --- | --- | --- | --- | --- | --- | --- | --- | --- | --- |
|  |  |  |  |  |  |  |  |  |  |  |
|  |  | **SCP vs. WMT** | | | **LZT vs. WMT** | | | **SCP vs. LZT** | | |
| **Measurement** | | **Treatment effect (95% CI)** | **Sig.** | **Cohens d’** | **Treatment effect (95% CI)** | **Sig.** | **Cohens d’** | **Treatment effect (95% CI)** | **Sig.** | **Cohens d’** |
| Digit Span- forward**^↑a^** | | **-1.45 (-2.48 to -0.42)** | **0.006** | **-0.48** | **-1.47 (-2.52 to -0.43)** | **0.006** | **-0.47** | 0.02 (-0.88 to 0.92) | 0.961 | 0.01 |
| Digit Span- backward**^↑a^** | | -0.87 (-2.18 to 0.43) | 0.189 | -0.30 | -1.06 (-2.24 to 0.13) | 0.081 | -0.36 | 0.20 (-0.90 to 1.31) | 0.716 | 0.07 |
| Number Letter Sequences**^↑a^** | | -0.41 (-1.28 to 0.45) | 0.343 | -0.16 | -0.43 (-1.19 to 0.34) | 0.273 | -0.17 | 0.03 (-0.79 to 0.85) | 0.939 | 0.01 |
| Block Tapping - forward**^↑b^** | | **-1.94 (-2.70 to -1.18)** | **0.000** | **-0.96** | **-1.96 (-2.70 to -1.22)** | **0.000** | **-0.99** | 0.01 (-0.70 to 0.73) | 0.969 | 0.01 |
| Block Tapping - backward**^↑b^** | | **-1.15 (-1.98 to -0.32)** | **0.007** | **-0.51** | **-1.39 (-2.30 to -0.48)** | **0.003** | **-0.61** | 0.22 (-0.64 to 1.07) | 0.618 | 0.10 |
| Telephone task - BSE**^↓^** | | 0.89 (-2.45 to 4.23) | 0.597 | 0.10 | 2.42 (-1.47 to 6.32) | 0.221 | 0.25 | -1.51 (-5.19 to 2.17) | 0.417 | -0.15 |
| Telephone task - WSE**^↓^** | | 0.48 (-0.28 to 1.24) | 0.213 | 0.28 | 0.43 (-0.39 to 1.24) | 0.304 | 0.23 | 0.05 (-0.70 to 0.80) | 0.890 | 0.04 |
| CPT-II - Omissions**^↓^** | | -3.63 (-8.85 to 1.59) | 0.171 | -0.30 | -2.38 (-7.75 to 2.99) | 0.380 | -0.21 | -1.31 (-6.15 to 3.52) | 0.591 | -0.13 |
| CPT-II - Commissions**^↓^** | | -2.97 (-6.30 to 0.36) | 0.080 | -0.30 | -0.65 (-3.94 to 2.63) | 0.693 | -0.06 | -2.30 (-5.43 to 0.84) | 0.149 | -0.22 |
| CPT-II - Hit RT**^↓^** | | -0.38 (-3.85 to 3.09) | 0.829 | -0.04 | -1.10 (-4.41 to 2.22) | 0.512 | -0.11 | 0.73 (-2.67 to 4.13) | 0.671 | 0.07 |
| CPT-II - Hit RT SE**^↓^** | | -2.71 (-6.24 to 0.83) | 0.132 | -0.30 | -3.13 (-7.05 to 0.78) | 0.115 | -0.34 | 0.44 (-2.87 to 3.74) | 0.794 | 0.05 |
| CPT-II - ADHD-index**^↓c^** | | -5.56 (-12.74 to 1.62) | 0.128 | -0.31 | -4.13 (-11.16 to 2.90) | 0.247 | -0.24 | -1.49 (-7.91 to 4.92) | 0.645 | -0.09 |
| Tapping - CoV**^↓^** | | 3.29 (-0.69 to 7.28) | 0.104 | 0.28 | 2.34 (-1.62 to 6.30) | 0.244 | 0.20 | 0.97 (-1.71 to 3.64) | 0.477 | 0.15 |
| TA 400ms – Hit rate **^↑^** | | 0.00 (-0.07 to 0.06) | 0.951 | -0.01 | -0.01 (-0.07 to 0.05) | 0.706 | -0.08 | 0.01 (-0.05 to 0.07) | 0.751 | 0.07 |
| TA 400ms – Too Early**^↓^** | | -0.01 (-0.07 to 0.04) | 0.575 | -0.12 | 0.01 (-0.04 to 0.05) | 0.823 | 0.05 | -0.02 (-0.07 to 0.03) | 0.423 | -0.17 |
| TA 2000ms – Hit rate**^↑^** | | -0.04 (-0.13 to 0.05) | 0.345 | -0.15 | **-0.09 (-0.18 to -0.01)** | **0.034** | **-0.39** | 0.05 (-0.03 to 0.13) | 0.221 | 0.21 |
| TA 2000ms – Too Early**^↓^** | | 0.00 (-0.09 to 0.09) | 0.963 | 0.01 | **0.09 (0.01 to 0.17)** | **0.037** | **0.39** | **-0.09 (-0.17 to 0.00)** | **0.040** | **-0.37** |
| **Note**:**^↓^=**Negative values favor the first intervention; **^↑^=**Positive values favor the first intervention; BSE = Between-search-errors (raw score); WSE = Within-search-errors (raw score); CPT-II = Conners’ Continuous Performance task (t-scores; 50±10); CoV = Coefficient of Variability (SD/mean tapping rate x 100); TA = Time Anticipation (percentages; max score 1.00); RT = Reaction Time; SE = Standard Error; ^a^ = Scale scores (10±3) ; ^b^ = raw scores (max score 14) ; ^c^ = percentages (max score 100) ; Significant results are **bold** | | | | | | | | | | |

| **Supplement Table S3** Comparison between the active interventions from baseline to 6-month follow-up | | | | | | | | | | |
| --- | --- | --- | --- | --- | --- | --- | --- | --- | --- | --- |
|  |  |  |  |  |  |  |  |  |  |  |
|  |  | **SCP vs. WMT** | | | **LZT vs. WMT** | | | **SCP vs. LZT** | | |
| **Measurement** | | **Treatment effect (95% CI)** | **Sig.** | **Cohens d’** | **Treatment effect (95% CI)** | **Sig.** | **Cohens d’** | **Treatment effect (95% CI)** | **Sig.** | **Cohens d’** |
| Digit Span- forward**^↑a^** | | -0.44 (-1.43 to 0.55) | 0.376 | -0.15 | **-1.53 (-2.55 to -0.50)** | **0.004** | **-0.49** | **1.12 (0.19 to 2.05)** | **0.019** | **0.44** |
| Digit Span- backward**^↑a^** | | -1.15 (-2.37 to 0.06) | 0.062 | -0.39 | -0.54 (-1.70 to 0.61) | 0.353 | -0.18 | -0.59 (-1.78 to 0.61) | 0.333 | -0.19 |
| Number Letter Sequences**^↑a^** | | -0.82 (-1.93 to 0.30) | 0.150 | -0.32 | -0.43 (-1.44 to 0.57) | 0.397 | -0.17 | -0.37 (-1.32 to 0.58) | 0.444 | -0.14 |
| Block Tapping - forward**^↑b^** | | **-1.60 (-2.35 to -0.85)** | **0.000** | **-0.79** | **-1.92 (-2.75 to -1.10)** | **0.000** | **-0.97** | 0.32 (-0.48 to 1.12) | 0.430 | 0.16 |
| Block Tapping - backward**^↑b^** | | **-0.96 (-1.85 to -0.07)** | **0.035** | **-0.43** | **-0.96 (-1.89 to -0.02)** | **0.045** | **-0.42** | -0.02 (-0.92 to 0.88) | 0.960 | -0.01 |
| Telephone task - BSE**^↓^** | | 0.29 (-3.34 to 3.92) | 0.874 | 0.03 | 0.00 (-4.10 to 4.10) | 1.000 | 0.00 | 0.33 (-3.55 to 4.21) | 0.867 | 0.03 |
| Telephone task - WSE**^↓^** | | 0.52 (-0.25 to 1.29) | 0.183 | 0.30 | 0.08 (-0.76 to 0.93) | 0.849 | 0.04 | 0.44 (-0.29 to 1.16) | 0.238 | 0.31 |
| CPT-II - Omissions**^↓^** | | -1.38 (-5.81 to 3.04) | 0.536 | -0.11 | -1.93 (-5.77 to 1.90) | 0.319 | -0.17 | 0.59 (-2.81 to 4.00) | 0.730 | 0.06 |
| CPT-II - Commissions**^↓^** | | 0.40 (-3.21 to 4.01) | 0.827 | 0.04 | -1.84 (-5.51 to 1.82) | 0.320 | -0.17 | 2.23 (-1.21 to 5.66) | 0.202 | 0.21 |
| CPT-II - Hit RT**^↓^** | | -1.19 (-5.08 to 2.70) | 0.545 | -0.11 | -0.91 (-4.75 to 2.93) | 0.641 | -0.09 | -0.25 (-3.85 to 3.34) | 0.890 | -0.03 |
| CPT-II - Hit RT SE**^↓^** | | 0.07 (-3.34 to 3.48) | 0.967 | 0.01 | -1.30 (-4.99 to 2.39) | 0.486 | -0.14 | 1.51 (-2.05 to 5.06) | 0.403 | 0.16 |
| CPT-II - ADHD-index**^↓c^** | | -1.03 (-8.80 to 6.74) | 0.793 | -0.06 | -2.13 (-9.51 to 5.25) | 0.568 | -0.12 | 1.01 (-5.59 to 7.62) | 0.761 | 0.06 |
| Tapping - CoV**^↓^** | | 3.54 (-0.04 to 7.12) | 0.053 | 0.30 | **2.86 (0.12 to 5.60)** | **0.041** | **0.25** | 0.65 (-2.41 to 3.72) | 0.674 | 0.10 |
| TA 400ms – Hit rate **^↑^** | | -0.03 (-0.10 to 0.04) | 0.420 | -0.17 | -0.04 (-0.10 to 0.02) | 0.171 | -0.28 | 0.01 (-0.05 to 0.08) | 0.628 | 0.10 |
| TA 400ms – Too Early**^↓^** | | 0.03 (-0.03 to 0.08) | 0.322 | 0.24 | 0.04 (-0.01 to 0.09) | 0.092 | 0.37 | -0.01 (-0.07 to 0.04) | 0.612 | -0.11 |
| TA 2000ms – Hit rate**^↑^** | | -0.04 (-0.15 to 0.06) | 0.410 | -0.16 | **-0.11 (-0.20 to -0.01)** | **0.024** | **-0.44** | 0.06 (-0.03 to 0.15) | 0.202 | 0.24 |
| TA 2000ms – Too Early**^↓^** | | 0.01 (-0.09 to 0.11) | 0.866 | 0.03 | 0.08 (0.00 to 0.17) | 0.057 | 0.37 | -0.07 (-0.16 to 0.02) | 0.116 | -0.31 |
| **Note**:**^↓^=**Negative values favor the first intervention; **^↑^=**Positive values favor the first intervention; BSE = Between-search-errors (raw score); WSE = Within-search-errors (raw score); CPT-II = Conners’ Continuous Performance task (t-scores; 50±10); CoV = Coefficient of Variability (SD/mean tapping rate x 100); TA = Time Anticipation (percentages; max score 1.00); RT = Reaction Time; SE = Standard Error; ^a^ = Scale scores (10±3) ; ^b^ = raw scores (max score 14) ; ^c^ = percentages (max score 100) ; Significant results are **bold** | | | | | | | | | | |

| **Supplement Table S4a** Baseline characteristics of participants with ADHD combined presentation | | | | | |
| --- | --- | --- | --- | --- | --- |
|  | | **SCP** | **LZT** | **WMT** | **TAU** |
| N= 129 | | 31 | 31 | 40 | 27 |
| Age in years M (SD) | | 11.62 (2.1) | 12.19 (2.2) | 12.09 (2.5) | 11.70 (2.7) |
| Male:Female | | 21:10 | 23:8 | 32:8 | 21:6 |
| IQ. M (SD) | 107.3 (15.0) | | 101.6 (12.8) | 100.6 (15.2) | 104.6 (14.7) |
| ADHD severity –  Teacher t-value M (SD) | | 66.21 (13.9) | 67.77 (14.6) | 65.97 (15.7) | 69.26 (15.0) |
| ADHD severity –  Parent t-value M (SD) | | 84.74 (10.9) | 85.06 (7.7) | 83.44 (10.0) | 84.41 (9.7) |
| ADHD severity –  Self t-value M (SD) | | 72.97 (16.1) | 69.06 (15.4) | 72.72 (16.9) | 71.89 (15.3) |
| ASD comorbid ASD. n (%) | | 2 (6%) | 6 (19%) | 5 (13%) | 5 (19%) |
| Comorbid psychiatric disorder^e^. n (%) | | 15 (48%) | 13 (42%) | 17 (43%) | 10 (37%) |
| ADHD –medication.  n (%) | | 15 (48%) | 21 (68%) | 25 (63%) | 18 (67%) |
| *Note*. ^a^ Childhood Autism (CA) x1. Atypical autism (AA) x 2. Asperger syndrome (AS) x 5;  ^b^ AA x1. AS x6;  ^c^ AA x3. AS x 4;  ^d^ CA x3. AA x4. AS x5;  ^e^ Include Mood Disorders. Anxiety disorders. Oppositional Defiant Disorder. Sleeping Disorders. Learning Disorders and Speech Disorders; | | | | | |

| **Supplement Table S4b** Baseline characteristics of participants with ADHD predominantly inattentive presentation | | | | | |
| --- | --- | --- | --- | --- | --- |
|  | | **SCP** | **LZT** | **WMT** | **TAU** |
| N= 73 | | 20 | 19 | 11 | 23 |
| Age in years. M (SD) | | 13.49 (3.03) | 12.77 (2.47) | 14.50 (2.86) | 12.81 (1.85) |
| Male:Female | | 17:3 | 14:5 | 10:1 | 15:8 |
| IQ. M (SD) | 101.4 (15.5) | | 102.2 (13.1) | 107.0 (18.1) | 95.5 (13.6) |
| ADHD severity – Teacher t-value M (SD) | | 57.39 (10.7) | 62.41 (15.2) | 58.50 (13.7) | 63.50 (13.8) |
| ADHD severity –  Parent t-value. M (SD) | | 73.95 (15.7) | 79.21 (10.4) | 73.82 (18.7) | 80.91 (12.1) |
| ADHD severity –  Self t-value. M (SD) | | 63.45 (14.8) | 63.95 (14.5) | 70.64 (16.5) | 67.82 (16.5) |
| ASD comorbid ASD.  n (%) | | 5 (25%) | 3 (16%) | 4 (36%) | 10 (43%) |
| Comorbid psychiatric disorder^e^. n (%) | | 5 (25%) | 3 (16%) | 4 (36%) | 10 (43%) |
| ADHD –medication.  n (%) | | 10 (50%) | 11 (58%) | 8 (73%) | 17 (74%) |
| *Note*. ^a^ Childhood Autism (CA) x1. Atypical autism (AA) x 2. Asperger syndrome (AS) x 5;  ^b^ AA x1. AS x6;  ^c^ AA x3. AS x 4;  ^d^ CA x3. AA x4. AS x5;  ^e^ Include Mood Disorders. Anxiety disorders. Oppositional Defiant Disorder. Sleeping Disorders. Learning Disorders and Speech Disorders; | | | | | |

| **Supplement Table S5a** Results for active interventions compared to treatment-as-usual from baseline to posttreatment, for participants with ADHD combined presentation | | | | | | | | | | |
| --- | --- | --- | --- | --- | --- | --- | --- | --- | --- | --- |
|  |  |  |  |  |  |  |  |  |  |  |
|  |  | **SCP vs. TAU** | | | **LZT vs. TAU** | | | **WMT vs. TAU** | | |
| **Measurement** | | **Treatment effect (95% CI)** | **Sig.** | **Cohens d’** | **Treatment effect (95% CI)** | **Sig.** | **Cohens d’** | **Treatment effect (95% CI)** | **Sig.** | **Cohens d’** |
| Digit Span- forward**^↑^** | | 0.16 (-1.02 to 1.34) | 0.789 | 0.08 | -0.15 (-1.46 to 1.16) | 0.819 | -0.08 | **1.40 (0.10 to 2.70)** | **0.035** | **0.49** |
| Digit Span- backward**^↑^** | | 0.22 (-1.27 to 1.71) | 0.770 | 0.07 | 0.09 (-1.31 to 1.50) | 0.896 | 0.04 | 0.71 (-0.88 to 2.30) | 0.376 | 0.27 |
| Number Letter Sequences**^↑^** | | -1.30 (-2.40 to -0.21) | 0.021 | -0.49 | **-1.10 (-2.19 to -0.02)** | **0.046** | **-0.39** | -0.59 (-1.66 to 0.47) | 0.271 | -0.22 |
| Block Tapping - forward**^↑^** | | -0.47 (-1.47 to 0.52) | 0.344 | -0.24 | -0.89 (-1.81 to 0.04) | 0.059 | -0.44 | **1.28 (0.39 to 2.17)** | **0.005** | **0.66** |
| Block Tapping - backward**^↑^** | | -0.03 (-0.99 to 0.94) | 0.957 | -0.01 | -0.09 (-1.27 to 1.08) | 0.873 | -0.04 | 0.92 (-0.08 to 1.92) | 0.071 | 0.44 |
| Telephone task - BSE**^↓^** | | -0.58 (-5.67 to 4.52) | 0.822 | -0.06 | -1.38 (-6.87 to 4.11) | 0.616 | -0.13 | -1.23 (-6.42 to 3.96) | 0.637 | -0.13 |
| Telephone task - WSE**^↓^** | | -0.04 (-1.17 to 1.10) | 0.946 | -0.53 | -1.23 (-3.07 to 0.62) | 0.190 | -0.63 | -1.21 (-2.94 to 0.52) | 0.169 | -0.64 |
| CPT-II - Omissions**^↓^** | | -0.98 (-6.35 to 4.39) | 0.716 | -0.08 | 0.38 (-5.05 to 5.80) | 0.889 | 0.04 | 3.82 (-2.25 to 9.89) | 0.212 | 0.29 |
| CPT-II - Commissions**^↓^** | | -2.35 (-6.31 to 1.62) | 0.241 | -0.24 | 0.24 (-3.72 to 4.20) | 0.903 | 0.02 | 0.10 (-3.92 to 4.12) | 0.962 | 0.01 |
| CPT-II - Hit RT**^↓^** | | 2.39 (-2.33 to 7.11) | 0.316 | 0.21 | 3.24 (-0.99 to 7.47) | 0.130 | 0.31 | 3.50 (-0.67 to 7.67) | 0.098 | 0.31 |
| CPT-II - Hit RT SE**^↓^** | | -0.94 (-4.94 to 3.06) | 0.639 | -0.10 | 1.16 (-3.50 to 5.82) | 0.619 | 0.11 | 1.83 (-2.65 to 6.32) | 0.417 | 0.19 |
| CPT-II - ADHD-index**^↓^** | | 1.60 (-7.64 to 10.84) | 0.730 | 0.08 | 6.68 (-2.24 to 15.60) | 0.139 | 0.35 | 7.36 (-2.43 to 17.14) | 0.138 | 0.34 |
| Tapping - CoV**^↓^** | | 3.24 (-1.42 to 7.90) | 0.170 | 0.38 | 3.37 (-0.59 to 7.33) | 0.094 | 0.45 | -0.76 (-6.92 to 5.40) | 0.806 | -0.05 |
| TA 400ms – Hit rate **^↑^** | | 0.02 (-0.06 to 0.10) | 0.607 | 0.15 | 0.00 (-0.08 to 0.08) | 0.988 | 0.00 | 0.00 (-0.08 to 0.09) | 0.949 | 0.02 |
| TA 400ms – Too Early**^↓^** | | -0.04 (-0.11 to 0.02) | 0.203 | -0.37 | -0.02 (-0.08 to 0.04) | 0.508 | -0.20 | -0.01 (-0.07 to 0.05) | 0.839 | -0.06 |
| TA 2000ms – Hit rate**^↑^** | | -0.04 (-0.17 to 0.09) | 0.550 | -0.14 | -0.04 (-0.17 to 0.09) | 0.513 | -0.16 | 0.01 (-0.12 to 0.13) | 0.930 | 0.02 |
| TA 2000ms – Too Early**^↓^** | | 0.00 (-0.14 to 0.13) | 0.947 | -0.02 | 0.04 (-0.08 to 0.16) | 0.521 | 0.16 | 0.00 (-0.12 to 0.12) | 0.965 | 0.01 |
| **Note**:**^↓^=**Negative values favor the first intervention; **^↑^=**Positive values favor the first intervention; BSE = Between-search-errors (raw score); WSE = Within-search-errors (raw score); CPT-II = Conners’ Continuous Performance task (t-scores; 50±10); CoV = Coefficient of Variability (SD/mean tapping rate x 100); TA = Time Anticipation (percentages; max score 1.00); RT = Reaction Time; SE = Standard Error; ^a^ = Scale scores (10±3) ; ^b^ = raw scores (max score 14) ; ^c^ = percentages (max score 100) ; Significant results are **bold** | | | | | | | | | | |

| **Supplement Table S5b** Results for active interventions compared to treatment-as-usual from baseline to posttreatment, for participants with ADHD predominantly inattentive presentation | | | | | | | | | | |
| --- | --- | --- | --- | --- | --- | --- | --- | --- | --- | --- |
|  |  |  |  |  |  |  |  |  |  |  |
|  |  | **SCP vs. TAU** | | | **LZT vs. TAU** | | | **WMT vs. TAU** | | |
| **Measurement** | | **Treatment effect (95% CI)** | **Sig.** | **Cohens d’** | **Treatment effect (95% CI)** | **Sig.** | **Cohens d’** | **Treatment effect (95% CI)** | **Sig.** | **Cohens d’** |
| Digit Span- forward**^↑a^** | | -0.85 (-2.32 to 0.61) | 0.246 | -0.30 | -0.37 (-1.57 to 0.83) | 0.537 | -0.12 | 1.17 (-0.81 to 3.15) | 0.240 | 0.36 |
| Digit Span- backward**^↑a^** | | -1.11 (-2.95 to 0.72) | 0.229 | -0.43 | **-1.42 (-2.82 to -0.02)** | **0.047** | **-0.44** | 0.73 (-1.33 to 2.78) | 0.476 | 0.25 |
| Number Letter Sequences**^↑a^** | | 0.13 (-1.57 to 1.82) | 0.880 | 0.04 | -0.35 (-1.84 to 1.15) | 0.642 | -0.12 | 0.12 (-1.85 to 2.08) | 0.902 | 0.04 |
| Block Tapping - forward**^↑b^** | | 0.04 (-1.00 to 1.07) | 0.944 | 0.01 | 0.64 (-0.41 to 1.69) | 0.225 | 0.29 | **2.59 (1.15 to 4.04)** | **0.001** | **1.03** |
| Block Tapping - backward**^↑b^** | | 0.18 (-1.40 to 1.77) | 0.817 | 0.07 | -0.30 (-1.87 to 1.27) | 0.706 | -0.14 | **1.82 (-0.21 to 3.86)** | **0.078** | **0.69** |
| Telephone task - BSE**^↓^** | | -2.64 (-8.49 to 3.20) | 0.365 | -0.27 | 2.87 (-4.25 to 10.00) | 0.421 | 0.27 | -4.75 (-11.94 to 2.43) | 0.187 | -0.49 |
| Telephone task - WSE**^↓^** | | -0.29 (-1.70 to 1.12) | 0.683 | -0.17 | -0.07 (-1.65 to 1.51) | 0.933 | -0.04 | -1.53 (-3.44 to 0.37) | 0.111 | -0.61 |
| CPT-II - Omissions**^↓^** | | 2.44 (-4.36 to 9.24) | 0.474 | 0.28 | 3.07 (-3.91 to 10.06) | 0.379 | 0.38 | 2.69 (-4.70 to 10.08) | 0.466 | 0.35 |
| CPT-II - Commissions**^↓^** | | 0.99 (-3.80 to 5.77) | 0.680 | 0.11 | 2.83 (-1.67 to 7.32) | 0.211 | 0.25 | 5.22 (-0.44 to 10.89) | 0.070 | 0.50 |
| CPT-II - Hit RT**^↓^** | | -0.61 (-5.02 to 3.81) | 0.782 | -0.07 | -3.69 (-8.34 to 0.95) | 0.116 | -0.36 | -3.14 (-8.23 to 1.96) | 0.219 | -0.35 |
| CPT-II - Hit RT SE**^↓^** | | 2.59 (-2.16 to 7.33) | 0.278 | 0.30 | -1.71 (-6.61 to 3.20) | 0.486 | -0.19 | 6.11 (-0.85 to 13.06) | 0.083 | 0.78 |
| CPT-II - ADHD-index**^↓c^** | | 2.41 (-6.71 to 11.52) | 0.598 | 0.16 | -1.94 (-10.67 to 6.78) | 0.656 | -0.13 | 8.87 (-1.77 to 19.52) | 0.100 | 0.70 |
| Tapping - CoV**^↓^** | | 0.70 (-2.63 to 4.02) | 0.675 | 0.11 | -2.00 (-5.99 to 1.99) | 0.317 | -0.28 | -1.42 (-5.55 to 2.71) | 0.492 | -0.24 |
| TA 400ms – Hit rate **^↑^** | | -0.02 (-0.12 to 0.09) | 0.752 | -0.11 | -0.01 (-0.11 to 0.09) | 0.848 | -0.06 | 0.03 (-0.09 to 0.15) | 0.599 | 0.20 |
| TA 400ms – Too Early**^↓^** | | 0.02 (-0.06 to 0.11) | 0.586 | 0.17 | 0.04 (-0.05 to 0.13) | 0.373 | 0.30 | -0.02 (-0.13 to 0.09) | 0.726 | -0.13 |
| TA 2000ms – Hit rate**^↑^** | | -0.03 (-0.16 to 0.10) | 0.614 | -0.13 | **-0.16 (-0.29 to -0.03)** | **0.018** | **-0.77** | 0.02 (-0.15 to 0.19) | 0.812 | 0.08 |
| TA 2000ms – Too Early**^↓^** | | -0.02 (-0.14 to 0.11) | 0.781 | -0.07 | **0.13 (0.00 to 0.26)** | **0.050** | **0.66** | -0.07 (-0.23 to 0.09) | 0.367 | -0.30 |
| **Note**:**^↓^=**Negative values favor the first intervention; **^↑^=**Positive values favor the first intervention; BSE = Between-search-errors (raw score); WSE = Within-search-errors (raw score); CPT-II = Conners’ Continuous Performance task (t-scores; 50±10); CoV = Coefficient of Variability (SD/mean tapping rate x 100); TA = Time Anticipation (percentages; max score 1.00); RT = Reaction Time; SE = Standard Error; ^a^ = Scale scores (10±3) ; ^b^ = raw scores (max score 14) ; ^c^ = percentages (max score 100) ; Significant results are **bold** | | | | | | | | | | |

| **Supplement Table S6a** Results for active interventions compared to treatment-as-usual from baseline to 6-month follow-up, for participants with ADHD combined presentation | | | | | | | | | | |
| --- | --- | --- | --- | --- | --- | --- | --- | --- | --- | --- |
|  |  |  |  |  |  |  |  |  |  |  |
|  |  | **SCP vs. TAU** | | | **LZT vs. TAU** | | | **WMT vs. TAU** | | |
| **Measurement** | | **Treatment effect (95% CI)** | **Sig.** | **Cohens d’** | **Treatment effect (95% CI)** | **Sig.** | **Cohens d’** | **Treatment effect (95% CI)** | **Sig.** | **Cohens d’** |
| Digit Span- forward**^↑a^** | | 1.12 (-0.09 to 2.33) | 0.068 | 0.57 | -0.78 (-2.12 to 0.55) | 0.246 | -0.40 | 0.92 (-0.31 to 2.14) | 0.139 | 0.32 |
| Digit Span- backward**^↑a^** | | -0.89 (-2.47 to 0.69) | 0.265 | -0.30 | 0.36 (-1.21 to 1.93) | 0.651 | 0.14 | 0.56 (-0.94 to 2.06) | 0.461 | 0.22 |
| Number Letter Sequences**^↑a^** | | -1.16 (-2.47 to 0.14) | 0.080 | -0.44 | -0.90 (-2.20 to 0.41) | 0.175 | -0.32 | -0.15 (-1.55 to 1.25) | 0.832 | -0.06 |
| Block Tapping - forward**^↑b^** | | -0.22 (-1.17 to 0.72) | 0.641 | -0.11 | **-1.55 (-2.56 to -0.53)** | **0.003** | **-0.77** | **1.04 (0.15 to 1.92)** | **0.022** | **0.53** |
| Block Tapping - backward**^↑b^** | | 0.22 (-0.75 to 1.19) | 0.645 | 0.12 | 0.36 (-0.72 to 1.45) | 0.503 | 0.17 | **1.12 (0.13 to 2.10)** | **0.027** | 0.**54** |
| Telephone task - BSE**^↓^** | | 4.26 (-0.40 to 8.92) | 0.073 | 0.44 | 2.98 (-2.35 to 8.31) | 0.268 | 0.28 | 4.03 (-0.90 to 8.96) | 0.108 | 0.43 |
| Telephone task - WSE**^↓^** | | -0.97 (-2.78 to 0.84) | 0.287 | -0.02 | -0.43 (-1.63 to 0.77) | 0.476 | -0.22 | -0.29 (-1.43 to 0.86) | 0.618 | -0.15 |
| CPT-II - Omissions**^↓^** | | -2.83 (-10.77 to 5.10) | 0.478 | -0.23 | -5.70 (-12.98 to 1.58) | 0.122 | -0.54 | -4.86 (-12.51 to 2.79) | 0.209 | -0.37 |
| CPT-II - Commissions**^↓^** | | 0.37 (-4.24 to 4.98) | 0.873 | 0.04 | -3.34 (-8.10 to 1.42) | 0.166 | -0.32 | 0.35 (-4.32 to 5.02) | 0.882 | 0.03 |
| CPT-II - Hit RT**^↓^** | | 0.52 (-3.82 to 4.85) | 0.813 | 0.04 | 2.42 (-1.75 to 6.59) | 0.250 | 0.23 | 1.54 (-3.25 to 6.33) | 0.524 | 0.13 |
| CPT-II - Hit RT SE**^↓^** | | -1.12 (-6.01 to 3.77) | 0.649 | -0.11 | -2.47 (-7.38 to 2.44) | 0.319 | -0.24 | -1.96 (-6.75 to 2.82) | 0.417 | -0.21 |
| CPT-II - ADHD-index**^↓c^** | | 1.40 (-9.06 to 11.86) | 0.790 | 0.07 | 0.01 (-9.17 to 9.19) | 0.998 | 0.00 | 0.00 (-10.67 to 10.67) | 1.000 | 0.00 |
| Tapping - CoV**^↓^** | | 2.96 (-2.70 to 8.61) | 0.298 | 0.35 | 1.75 (-2.13 to 5.63) | 0.314 | 0.23 | -2.29 (-6.87 to 2.30) | 0.321 | -0.16 |
| TA 400ms – Hit rate **^↑^** | | -0.04 (-0.12 to 0.05) | 0.395 | -0.26 | -0.03 (-0.11 to 0.05) | 0.472 | -0.21 | 0.00 (-0.09 to 0.09) | 0.991 | 0.00 |
| TA 400ms – Too Early**^↓^** | | 0.00 (-0.08 to 0.07) | 0.927 | -0.03 | 0.01 (-0.06 to 0.07) | 0.875 | 0.05 | -0.03 (-0.09 to 0.04) | 0.461 | -0.24 |
| TA 2000ms – Hit rate**^↑^** | | -0.04 (-0.18 to 0.10) | 0.570 | -0.14 | -0.08 (-0.21 to 0.04) | 0.184 | -0.32 | -0.01 (-0.14 to 0.11) | 0.824 | -0.05 |
| TA 2000ms – Too Early**^↓^** | | 0.01 (-0.13 to 0.15) | 0.883 | 0.04 | 0.08 (-0.04 to 0.20) | 0.202 | 0.31 | 0.02 (-0.09 to 0.14) | 0.716 | 0.08 |
| **Note**:**^↓^=**Negative values favor the first intervention; **^↑^=**Positive values favor the first intervention; BSE = Between-search-errors (raw score); WSE = Within-search-errors (raw score); CPT-II = Conners’ Continuous Performance task (t-scores; 50±10); CoV = Coefficient of Variability (SD/mean tapping rate x 100); TA = Time Anticipation (percentages; max score 1.00); RT = Reaction Time; SE = Standard Error; ^a^ = Scale scores (10±3) ; ^b^ = raw scores (max score 14) ; ^c^ = percentages (max score 100) ; Significant results are **bold** | | | | | | | | | | |

| **Supplement Table S6b** Results for active interventions compared to treatment-as-usual from baseline to 6-month follow-up, for participants with ADHD predominantly inattentive presentation. | | | | | | | | | | |
| --- | --- | --- | --- | --- | --- | --- | --- | --- | --- | --- |
|  |  |  |  |  |  |  |  |  |  |  |
|  |  | **SCP vs. TAU** | | | **LZT vs. TAU** | | | **WMT vs. TAU** | | |
| **Measurement** | | **Treatment effect (95% CI)** | **Sig.** | **Cohens d’** | **Treatment effect (95% CI)** | **Sig.** | **Cohens d’** | **Treatment effect (95% CI)** | **Sig.** | **Cohens d’** |
| Digit Span- forward**^↑a^** | | -1.22 (-2.47 to 0.03) | 0.055 | -0.43 | -1.11 (-2.35 to 0.13) | 0.079 | -0.36 | 0.20 (-1.71 to 2.11) | 0.833 | 0.06 |
| Digit Span- backward**^↑a^** | | -0.94 (-2.82 to 0.95) | 0.324 | -0.36 | -1.33 (-3.02 to 0.36) | 0.120 | -0.41 | 0.21 (-1.86 to 2.28) | 0.841 | 0.07 |
| Number Letter Sequences**^↑a^** | | -0.45 (-2.47 to 1.57) | 0.655 | -0.15 | -0.01 (-1.71 to 1.68) | 0.986 | -0.01 | -0.02 (-2.40 to 2.36) | 0.985 | -0.01 |
| Block Tapping - forward**^↑b^** | | -0.61 (-1.86 to 0.64) | 0.331 | -0.25 | 0.68 (-0.71 to 2.07) | 0.331 | 0.31 | 1.44 (-0.34 to 3.21) | 0.110 | 0.57 |
| Block Tapping - backward**^↑b^** | | 0.49 (-1.20 to 2.18) | 0.559 | 0.20 | 0.31 (-1.38 to 1.99) | 0.716 | 0.14 | 1.62 (-0.59 to 3.83) | 0.146 | 0.62 |
| Telephone task - BSE**^↓^** | | 0.89 (-5.11 to 6.89) | 0.768 | 0.09 | 2.05 (-4.45 to 8.56) | 0.529 | 0.20 | 0.04 (-7.08 to 7.17) | 0.990 | 0.00 |
| Telephone task - WSE**^↓^** | | 0.03 (-1.35 to 1.42) | 0.961 | 0.02 | -0.46 (-1.98 to 1.06) | 0.547 | -0.26 | -1.13 (-3.12 to 0.86) | 0.259 | -0.45 |
| CPT-II - Omissions**^↓^** | | -2.35 (-6.87 to 2.17) | 0.302 | -0.27 | -0.45 (-4.27 to 3.37) | 0.814 | -0.06 | **6.47 (0.58 to 12.36)** | **0.032** | **0.84** |
| CPT-II - Commissions**^↓^** | | 0.26 (-5.31 to 5.82) | 0.926 | 0.03 | 0.55 (-4.77 to 5.86) | 0.836 | 0.05 | -1.97 (-9.07 to 5.14) | 0.576 | -0.19 |
| CPT-II - Hit RT**^↓^** | | 0.44 (-4.55 to 5.43) | 0.860 | 0.05 | -1.99 (-6.95 to 2.98) | 0.424 | -0.19 | 2.55 (-2.47 to 7.58) | 0.310 | 0.28 |
| CPT-II - Hit RT SE**^↓^** | | 0.04 (-4.81 to 4.89) | 0.987 | 0.00 | -1.89 (-7.66 to 3.88) | 0.513 | -0.21 | 1.45 (-4.00 to 6.89) | 0.592 | 0.19 |
| CPT-II - ADHD-index**^↓c^** | | -1.52 (-9.85 to 6.80) | 0.714 | -0.10 | -3.29 (-12.51 to 5.94) | 0.476 | -0.22 | 4.22 (-6.23 to 14.68) | 0.418 | 0.34 |
| Tapping - CoV**^↓^** | | -1.88 (-5.84 to 2.07) | 0.339 | -0.30 | -1.52 (-7.05 to 4.01) | 0.583 | -0.21 | -2.26 (-7.94 to 3.43) | 0.419 | -0.37 |
| TA 400ms – Hit rate **^↑^** | | 0.01 (-0.09 to 0.12) | 0.773 | 0.09 | -0.03 (-0.12 to 0.06) | 0.534 | -0.20 | 0.05 (-0.06 to 0.16) | 0.379 | 0.33 |
| TA 400ms – Too Early**^↓^** | | 0.00 (-0.09 to 0.09) | 0.952 | 0.02 | 0.02 (-0.06 to 0.11) | 0.620 | 0.16 | -0.06 (-0.17 to 0.05) | 0.258 | -0.43 |
| TA 2000ms – Hit rate**^↑^** | | 0.00 (-0.13 to 0.13) | 0.980 | 0.01 | -0.08 (-0.18 to 0.03) | 0.146 | -0.38 | 0.12 (-0.05 to 0.29) | 0.176 | 0.49 |
| TA 2000ms – Too Early**^↓^** | | -0.06 (-0.19 to 0.07) | 0.384 | -0.24 | 0.02 (-0.10 to 0.13) | 0.740 | 0.10 | -0.15 (-0.31 to 0.02) | 0.091 | -0.61 |
| **Note**:**^↓^=**Negative values favor the first intervention; **^↑^=**Positive values favor the first intervention; BSE = Between-search-errors (raw score); WSE = Within-search-errors (raw score); CPT-II = Conners’ Continuous Performance task (t-scores; 50±10); CoV = Coefficient of Variability (SD/mean tapping rate x 100); TA = Time Anticipation (percentages; max score 1.00); RT = Reaction Time; SE = Standard Error; ^a^ = Scale scores (10±3) ; ^b^ = raw scores (max score 14) ; ^c^ = percentages (max score 100) ; Significant results are **bold** | | | | | | | | | | |

| **Supplement Table S7a** Results for active interventions compared to treatment-as-usual from baseline to posttreatment, for medicated-participants. | | | | | | | | | | |
| --- | --- | --- | --- | --- | --- | --- | --- | --- | --- | --- |
|  |  | **SCP vs. TAU** | | | **LZT vs. TAU** | | | **WMT vs. TAU** | | |
| **Measurement** | | **Treatment effect (95% CI)** | **Sig.** | **Cohens d’** | **Treatment effect (95% CI)** | **Sig.** | **Cohens d’** | **Treatment effect (95% CI)** | **Sig.** | **Cohens d’** |
| Digit Span- forward**^↑a^** | | -0.16 (-1.25 to 0.93) | 0.768 | -0.07 | -0.69 (-2.39 to 1.01) | 0.417 | -0.23 | 0.75 (-0.42 to 1.91) | 0.208 | 0.23 |
| Digit Span- backward**^↑a^** | | 0.02 (-1.48 to 1.51) | 0.984 | 0.01 | -0.46 (-1.72 to 0.79) | 0.464 | -0.16 | 1.03 (-0.45 to 2.51) | 0.170 | 0.39 |
| Number Letter Sequences**^↑a^** | | -0.91 (-2.19 to 0.38) | 0.164 | -0.33 | **-1.17 (-2.25 to -0.10)** | **0.033** | **-0.43** | -0.46 (-1.65 to 0.73) | 0.447 | -0.16 |
| Block Tapping - forward**^↑b^** | | -0.33 (-1.25 to 0.60) | 0.482 | -0.15 | -0.28 (-1.16 to 0.60) | 0.523 | -0.13 | **1.55 (0.64 to 2.45)** | **0.001** | **0.72** |
| Block Tapping - backward**^↑b^** | | 0.29 (-0.82 to 1.40) | 0.603 | 0.13 | -0.29 (-1.42 to 0.84) | 0.610 | -0.14 | **1.44 (0.30 to 2.58)** | **0.014** | **0.65** |
| Telephone task - BSE**^↓^** | | -1.29 (-6.30 to 3.73) | 0.608 | -0.13 | 0.14 (-5.04 to 5.33) | 0.956 | 0.01 | -2.73 (-7.67 to 2.21) | 0.274 | -0.31 |
| Telephone task - WSE**^↓^** | | -1.04 (-2.96 to 0.87) | 0.281 | -0.56 | -0.93 (-2.74 to 0.87) | 0.305 | -0.46 | -1.06 (-2.79 to 0.67) | 0.225 | -0.56 |
| CPT-II - Omissions**^↓^** | | **-5.74 (-10.35 to -1.13)** | **0.016** | **-0.54** | -1.83 (-6.31 to 2.65) | 0.417 | -0.18 | 1.11 (-4.79 to 7.02) | 0.707 | 0.09 |
| CPT-II - Commissions**^↓^** | | -2.03 (-5.87 to 1.81) | 0.295 | -0.24 | -0.17 (-3.57 to 3.24) | 0.923 | -0.02 | 0.22 (-3.73 to 4.17) | 0.912 | 0.02 |
| CPT-II - Hit RT**^↓^** | | -1.44 (-5.18 to 2.30) | 0.444 | -0.14 | -1.07 (-4.93 to 2.79) | 0.582 | -0.11 | 0.32 (-3.40 to 4.05) | 0.863 | 0.03 |
| CPT-II - Hit RT SE**^↓^** | | -1.57 (-5.63 to 2.50) | 0.444 | -0.16 | 0.12 (-4.41 to 4.65) | 0.956 | 0.01 | 2.54 (-1.87 to 6.96) | 0.254 | 0.26 |
| CPT-II - ADHD-index**^↓c^** | | -4.28 (-12.77 to 4.21) | 0.317 | -0.24 | 1.88 (-6.01 to 9.77) | 0.636 | 0.11 | 5.51 (-2.84 to 13.86) | 0.192 | 0.29 |
| Tapping - CoV**^↓^** | | -0.83 (-3.86 to 2.20) | 0.588 | -0.11 | 0.53 (-2.31 to 3.38) | 0.710 | 0.08 | 0.01 (-0.09 to 0.12) | 0.772 | 0.00 |
| TA 400ms – Hit rate **^↑^** | | 0.01 (-0.08 to 0.10) | 0.877 | 0.05 | 0.03 (-0.04 to 0.11) | 0.393 | 0.22 | -0.01 (-0.09 to 0.08) | 0.870 | -0.04 |
| TA 400ms – Too Early**^↓^** | | -0.01 (-0.08 to 0.06) | 0.852 | -0.06 | -0.01 (-0.07 to 0.05) | 0.688 | -0.11 | 0.01 (-0.06 to 0.08) | 0.742 | 0.09 |
| TA 2000ms – Hit rate**^↑^** | | -0.05 (-0.16 to 0.06) | 0.381 | -0.18 | -0.06 (-0.18 to 0.05) | 0.275 | -0.25 | 0.00 (-0.11 to 0.11) | 0.978 | 0.01 |
| TA 2000ms – Too Early**^↓^** | | -0.02 (-0.13 to 0.09) | 0.742 | -0.07 | 0.03 (-0.08 to 0.14) | 0.575 | 0.13 | -0.04 (-0.14 to 0.07) | 0.500 | -0.15 |
| **Note**:**^↓^=**Negative values favor the first intervention; **^↑^=**Positive values favor the first intervention; BSE = Between-search-errors (raw score); WSE = Within-search-errors (raw score); CPT-II = Conners’ Continuous Performance task (t-scores; 50±10); CoV = Coefficient of Variability (SD/mean tapping rate x 100); TA = Time Anticipation (percentages; max score 1.00); RT = Reaction Time; SE = Standard Error; ^a^ = Scale scores (10±3) ; ^b^ = raw scores (max score 14) ; ^c^ = percentages (max score 100) ; Significant results are **bold** | | | | | | | | | | |

| **Supplement Table S7b** Results for active interventions compared to treatment-as-usual from baseline to posttreatment, for non-medicated participants. | | | | | | | | | | |
| --- | --- | --- | --- | --- | --- | --- | --- | --- | --- | --- |
|  |  | **SCP vs. TAU** | | | **LZT vs. TAU** | | | **WMT vs. TAU** | | |
| **Measurement** | | **Treatment effect (95% CI)** | **Sig.** | **Cohens d’** | **Treatment effect (95% CI)** | **Sig.** | **Cohens d’** | **Treatment effect (95% CI)** | **Sig.** | Cohens d’ |
| Digit Span- forward**^↑a^** | | -0.48 (-2.18 to 1.22) | 0.574 | -0.18 | 0.10 (-1.57 to 1.77) | 0.904 | 0.04 | 1.82 (-0.33 to 3.97) | 0.095 | 0.73 |
| Digit Span- backward**^↑a^** | | -0.59 (-2.49 to 1.30) | 0.532 | -0.18 | -0.46 (-1.72 to 0.79) | 0.464 | -0.25 | -0.30 (-2.45 to 1.85) | 0.780 | -0.11 |
| Number Letter Sequences**^↑a^** | | -0.36 (-1.88 to 1.15) | 0.627 | -0.12 | -0.01 (-1.54 to 1.52) | 0.988 | 0.00 | 0.10 (-1.36 to 1.56) | 0.889 | 0.04 |
| Block Tapping - forward**^↑b^** | | -0.44 (-1.71 to 0.83) | 0.489 | -0.21 | -0.34 (-1.49 to 0.80) | 0.541 | -0.17 | **1.82 (0.49 to 3.16)** | **0.009** | **0.93** |
| Block Tapping - backward**^↑b^** | | -0.03 (-1.46 to 1.40) | 0.970 | -0.01 | 0.19 (-1.51 to 1.88) | 0.823 | 0.09 | 0.97 (-0.57 to 2.51) | 0.212 | 0.41 |
| Telephone task - BSE**^↓^** | | 0.05 (-6.21 to 6.31) | 0.987 | 0.01 | 0.99 (-7.00 to 8.98) | 0.801 | 0.08 | -0.76 (-7.94 to 6.43) | 0.831 | -0.07 |
| Telephone task - WSE**^↓^** | | 0.06 (-1.07 to 1.20) | 0.909 | 0.04 | -0.29 (-1.41 to 0.83) | 0.595 | -0.19 | -1.25 (-2.61 to 0.10) | 0.068 | -0.52 |
| CPT-II - Omissions**^↓^** | | **9.83 (2.52 to 17.14)** | **0.010** | **0.86** | **8.78 (0.31 to 17.25)** | **0.043** | **0.93** | **10.67 (3.70 to 17.63)** | **0.004** | **1.12** |
| CPT-II - Commissions**^↓^** | | 2.15 (-3.04 to 7.34) | 0.407 | 0.20 | 5.10 (-0.68 to 10.87) | 0.082 | 0.46 | **6.23 (1.02 to 11.45)** | **0.021** | **0.55** |
| CPT-II - Hit RT**^↓^** | | 4.92 (-1.21 to 11.06) | 0.113 | 0.42 | 3.46 (-2.27 to 9.20) | 0.224 | 0.28 | 4.02 (-1.99 to 10.02) | 0.180 | 0.31 |
| CPT-II - Hit RT SE**^↓^** | | 3.24 (-1.57 to 8.05) | 0.181 | 0.36 | 0.47 (-4.71 to 5.65) | 0.854 | 0.05 | 4.80 (-1.77 to 11.37) | 0.147 | 0.54 |
| CPT-II - ADHD-index**^↓c^** | | 9.94 (-0.34 to 20.21) | 0.058 | 0.49 | 6.19 (-4.18 to 16.56) | 0.233 | 0.31 | 11.40 (-2.17 to 24.97) | 0.097 | 0.53 |
| Tapping - CoV**^↓^** | | **6.59 (0.64 to 12.54)** | **0.031** | **0.89** | 3.05 (-3.31 to 9.42) | 0.338 | 0.34 | -1.87 (-7.53 to 3.78) | 0.512 | -0.18 |
| TA 400ms – Hit rate **^↑^** | | -0.02 (-0.12 to 0.08) | 0.673 | -0.13 | -0.08 (-0.17 to 0.02) | 0.111 | -0.54 | 0.00 (-0.09 to 0.08) | 0.932 | -0.02 |
| TA 400ms – Too Early**^↓^** | | -0.01 (-0.09 to 0.08) | 0.853 | -0.06 | 0.05 (-0.04 to 0.13) | 0.296 | 0.38 | -0.01 (-0.09 to 0.08) | 0.870 | -0.06 |
| TA 2000ms – Hit rate**^↑^** | | -0.04 (-0.20 to 0.12) | 0.577 | -0.16 | -0.14 (-0.30 to 0.02) | 0.087 | -0.54 | 0.00 (-0.18 to 0.18) | 0.987 | 0.01 |
| TA 2000ms – Too Early**^↓^** | | 0.03 (-0.13 to 0.19) | 0.708 | 0.12 | **0.17 (0.01 to 0.32)** | **0.038** | **0.65** | 0.04 (-0.14 to 0.22) | 0.655 | 0.14 |
| **Note**:**^↓^=**Negative values favor the first intervention; **^↑^=**Positive values favor the first intervention; BSE = Between-search-errors (raw score); WSE = Within-search-errors (raw score); CPT-II = Conners’ Continuous Performance task (t-scores; 50±10); CoV = Coefficient of Variability (SD/mean tapping rate x 100); TA = Time Anticipation (percentages; max score 1.00); RT = Reaction Time; SE = Standard Error; ^a^ = Scale scores (10±3) ; ^b^ = raw scores (max score 14) ; ^c^ = percentages (max score 100) ; Significant results are **bold** | | | | | | | | | | |

| **Supplement Table S8a** Results for active interventions compared to treatment-as-usual from baseline to 6-month follow-up, for medicated-participants. | | | | | | | | | | |
| --- | --- | --- | --- | --- | --- | --- | --- | --- | --- | --- |
|  |  | **SCP vs. TAU** | | | **LZT vs. TAU** | | | **WMT vs. TAU** | | |
| **Measurement** | | **Treatment effect (95% CI)** | **Sig.** | **Cohens d’** | **Treatment effect (95% CI)** | **Sig.** | **Cohens d’** | **Treatment effect (95% CI)** | **Sig.** | **Cohens d’** |
| Digit Span- forward**^↑a^** | | 0.23 (-0.89 to 1.36) | 0.677 | 0.11 | 0.23 (-1.53 to 1.99) | 0.792 | -0.39 | 0.74 (-0.45 to 1.92) | 0.219 | 0.23 |
| Digit Span- backward**^↑a^** | | -0.69 (-2.24 to 0.87) | 0.382 | -0.28 | -0.73 (-2.25 to 0.79) | 0.343 | -0.25 | 0.46 (-0.96 to 1.87) | 0.522 | 0.17 |
| Number Letter Sequences**^↑a^** | | -1.27 (-2.84 to 0.30) | 0.111 | -0.47 | -1.18 (-2.53 to 0.18) | 0.088 | -0.43 | -0.08 (-1.44 to 1.29) | 0.912 | -0.03 |
| Block Tapping - forward**^↑b^** | | -0.15 (-1.21 to 0.90) | 0.774 | -0.07 | -0.70 (-1.72 to 0.33) | 0.179 | -0.33 | 0.96 (-0.05 to 1.98) | 0.063 | 0.45 |
| Block Tapping - backward**^↑b^** | | 0.67 (-0.56 to 1.89) | 0.281 | 0.30 | 0.48 (-0.65 to 1.61) | 0.402 | 0.22 | **1.60 (0.45 to 2.74)** | **0.007** | **0.72** |
| Telephone task - BSE**^↓^** | | **4.74 (0.40 to 9.08)** | **0.033** | **0.48** | 2.87 (-1.82 to 7.56) | 0.227 | 0.29 | 2.54 (-1.63 to 6.71) | 0.228 | 0.29 |
| Telephone task - WSE**^↓^** | | 0.14 (-0.97 to 1.26) | 0.797 | 0.08 | -0.51 (-1.64 to 0.62) | 0.371 | -0.25 | 0.10 (-0.96 to 1.15) | 0.858 | 0.05 |
| CPT-II - Omissions**^↓^** | | -5.86 (-12.97 to 1.26) | 0.105 | -0.55 | -6.17 (-12.60 to 0.27) | 0.060 | -0.61 | -3.47 (-10.21 to 3.27) | 0.307 | -0.27 |
| CPT-II - Commissions**^↓^** | | -0.78 (-5.04 to 3.47) | 0.715 | -0.09 | -2.95 (-6.90 to 1.00) | 0.141 | -0.28 | -1.83 (-6.33 to 2.66) | 0.420 | -0.19 |
| CPT-II - Hit RT**^↓^** | | -1.31 (-5.43 to 2.81) | 0.529 | -0.12 | 0.01 (-4.06 to 4.07) | 0.997 | 0.00 | 1.93 (-1.74 to 5.59) | 0.298 | 0.19 |
| CPT-II - Hit RT SE**^↓^** | | -2.12 (-6.97 to 2.74) | 0.388 | -0.22 | -1.70 (-6.73 to 3.34) | 0.505 | -0.17 | -0.68 (-5.19 to 3.83) | 0.766 | -0.07 |
| CPT-II - ADHD-index**^↓c^** | | -4.04 (-13.42 to 5.35) | 0.395 | -0.23 | -1.43 (-10.14 to 7.28) | 0.744 | -0.08 | -1.72 (-10.79 to 7.36) | 0.707 | -0.09 |
| Tapping - CoV**^↓^** | | -0.34 (-3.83 to 3.16) | 0.847 | -0.04 | 1.06 (-1.70 to 3.82) | 0.445 | 0.16 | 0.04 (-0.08 to 0.17) | 0.465 | 0.00 |
| TA 400ms – Hit rate **^↑^** | | -0.02 (-0.10 to 0.07) | 0.725 | -0.10 | -0.02 (-0.09 to 0.06) | 0.662 | -0.12 | -0.03 (-0.14 to 0.08) | 0.625 | -0.17 |
| TA 400ms – Too Early**^↓^** | | 0.01 (-0.06 to 0.09) | 0.704 | 0.12 | 0.01 (-0.05 to 0.07) | 0.726 | 0.09 | -0.03 (-0.09 to 0.04) | 0.423 | -0.21 |
| TA 2000ms – Hit rate**^↑^** | | 0.00 (-0.13 to 0.13) | 0.969 | -0.01 | -0.07 (-0.18 to 0.03) | 0.178 | -0.29 | 0.02 (-0.09 to 0.13) | 0.743 | 0.07 |
| TA 2000ms – Too Early**^↓^** | | -0.05 (-0.18 to 0.07) | 0.396 | -0.21 | 0.04 (-0.06 to 0.15) | 0.421 | 0.19 | -0.03 (-0.14 to 0.08) | 0.590 | -0.12 |
| **Note**:**^↓^=**Negative values favor the first intervention; **^↑^=**Positive values favor the first intervention; BSE = Between-search-errors (raw score); WSE = Within-search-errors (raw score); CPT-II = Conners’ Continuous Performance task (t-scores; 50±10); CoV = Coefficient of Variability (SD/mean tapping rate x 100); TA = Time Anticipation (percentages; max score 1.00); RT = Reaction Time; SE = Standard Error; ^a^ = Scale scores (10±3) ; ^b^ = raw scores (max score 14) ; ^c^ = percentages (max score 100) ; Significant results are **bold** | | | | | | | | | | |
| **Supplement Table S8b** Results for active interventions compared to treatment-as-usual from baseline to 6-month follow-up, for non-medicated participants. | | | | | | | | | | |
|  |  | **SCP vs. TAU** | | | **LZT vs. TAU** | | | **WMT vs. TAU** | | |
| **Measurement** | | **Treatment effect (95% CI)** | **Sig.** | **Cohens d’** | **Treatment effect (95% CI)** | **Sig.** | **Cohens d’** | **Treatment effect (95% CI)** | **Sig.** | **Cohens d’** |
| Digit Span- forward**^↑a^** | | -0.30 (-1.87 to 1.26) | 0.697 | -0.11 | -1.16 (-2.84 to 0.53) | 0.172 | -0.50 | -0.21 (-2.07 to 1.66) | 0.824 | -0.08 |
| Digit Span- backward**^↑a^** | | -1.12 (-3.13 to 0.89) | 0.269 | -0.34 | -0.73 (-2.25 to 0.79) | 0.343 | 0.08 | -0.16 (-2.28 to 1.96) | 0.880 | -0.06 |
| Number Letter Sequences**^↑a^** | | -0.43 (-2.18 to 1.33) | 0.627 | -0.14 | 0.48 (-1.10 to 2.06) | 0.544 | 0.16 | -0.08 (-2.56 to 2.41) | 0.950 | -0.03 |
| Block Tapping - forward**^↑b^** | | -0.73 (-1.84 to 0.38) | 0.190 | -0.35 | -0.69 (-2.17 to 0.79) | 0.354 | -0.34 | **1.76 (0.71 to 2.81)** | **0.002** | **0.90** |
| Block Tapping - backward**^↑b^** | | -0.32 (-1.66 to 1.01) | 0.626 | -0.17 | -0.01 (-1.74 to 1.71) | 0.988 | -0.01 | 0.68 (-0.98 to 2.34) | 0.407 | 0.29 |
| Telephone task - BSE**^↓^** | | 1.21 (-5.40 to 7.82) | 0.714 | 0.12 | 2.41 (-5.60 to 10.42) | 0.546 | 0.20 | 2.60 (-5.89 to 11.10) | 0.539 | 0.24 |
| Telephone task - WSE**^↓^** | | -0.39 (-1.92 to 1.15) | 0.614 | -0.24 | -0.14 (-1.99 to 1.70) | 0.875 | -0.09 | -1.85 (-3.99 to 0.29) | 0.088 | -0.76 |
| CPT-II - Omissions**^↓^** | | 2.96 (-2.67 to 8.60) | 0.295 | 0.26 | 1.95 (-3.30 to 7.19) | 0.456 | 0.21 | 3.86 (-4.50 to 12.21) | 0.352 | 0.41 |
| CPT-II - Commissions**^↓^** | | 2.60 (-3.66 to 8.86) | 0.404 | 0.24 | 0.54 (-6.93 to 8.00) | 0.884 | 0.05 | 3.53 (-3.23 to 10.30) | 0.291 | 0.31 |
| CPT-II - Hit RT**^↓^** | | 3.42 (-2.09 to 8.92) | 0.216 | 0.29 | 2.41 (-2.93 to 7.74) | 0.366 | 0.20 | 1.17 (-6.69 to 9.02) | 0.764 | 0.09 |
| CPT-II - Hit RT SE**^↓^** | | 0.98 (-3.60 to 5.57) | 0.665 | 0.11 | -2.62 (-7.42 to 2.17) | 0.273 | -0.28 | -0.86 (-6.86 to 5.14) | 0.773 | -0.10 |
| CPT-II - ADHD-index**^↓c^** | | 6.28 (-4.16 to 16.73) | 0.229 | 0.31 | 0.15 (-9.97 to 10.26) | 0.977 | 0.01 | 8.68 (-5.57 to 22.93) | 0.225 | 0.41 |
| Tapping - CoV**^↓^** | | 2.16 (-5.85 to 10.18) | 0.585 | 0.29 | -0.82 (-10.37 to 8.73) | 0.864 | -0.09 | -3.26 (-6.76 to 0.25) | 0.068 | -0.32 |
| TA 400ms – Hit rate **^↑^** | | -0.02 (-0.13 to 0.09) | 0.703 | -0.13 | -0.06 (-0.16 to 0.04) | 0.257 | -0.40 | -0.01 (-0.08 to 0.07) | 0.889 | -0.04 |
| TA 400ms – Too Early**^↓^** | | -0.01 (-0.11 to 0.10) | 0.878 | -0.06 | 0.02 (-0.08 to 0.12) | 0.643 | 0.20 | -0.03 (-0.14 to 0.08) | 0.625 | -0.25 |
| TA 2000ms – Hit rate**^↑^** | | -0.05 (-0.20 to 0.10) | 0.521 | -0.17 | -0.10 (-0.24 to 0.05) | 0.178 | -0.38 | 0.02 (-0.17 to 0.22) | 0.802 | 0.08 |
| TA 2000ms – Too Early**^↓^** | | 0.01 (-0.14 to 0.17) | 0.858 | 0.05 | 0.07 (-0.07 to 0.21) | 0.322 | 0.27 | -0.03 (-0.20 to 0.15) | 0.759 | -0.09 |
| **Note**:**^↓^=**Negative values favor the first intervention; **^↑^=**Positive values favor the first intervention; BSE = Between-search-errors (raw score); WSE = Within-search-errors (raw score); CPT-II = Conners’ Continuous Performance task (t-scores; 50±10); CoV = Coefficient of Variability (SD/mean tapping rate x 100); TA = Time Anticipation (percentages; max score 1.00); RT = Reaction Time; SE = Standard Error; ^a^ = Scale scores (10±3) ; ^b^ = raw scores (max score 14) ; ^c^ = percentages (max score 100) ; Significant results are **bold** | | | | | | | | | | |
